# Haplotype Variation of *Glu-D1* Locus and the Origin of *Glu-D1d* Allele Conferring Superior End-Use Qualities in Common Wheat

**DOI:** 10.1371/journal.pone.0074859

**Published:** 2013-09-30

**Authors:** Zhenying Dong, Yushuang Yang, Yiwen Li, Kunpu Zhang, Haijuan Lou, Xueli An, Lingli Dong, Yong Qiang Gu, Olin D. Anderson, Xin Liu, Huanju Qin, Daowen Wang

**Affiliations:** 1 The State Key Laboratory of Plant Cell and Chromosomal Engineering, Institute of Genetics and Developmental Biology, Chinese Academy of Sciences, Beijing, China; 2 Graduate University of Chinese Academy of Sciences, Beijing, China; 3 United States Department of Agriculture-Agricultural Research Service, Western Regional Research Center, Albany, California, United States of America; University of Iceland, Iceland

## Abstract

In higher plants, seed storage proteins (SSPs) are frequently expressed from complex gene families, and allelic variation of SSP genes often affects the quality traits of crops. In common wheat, the *Glu-D1* locus, encoding 1Dx and 1Dy SSPs, has multiple alleles. The *Glu-D1d* allele frequently confers superior end-use qualities to commercial wheat varieties. Here, we studied the haplotype structure of *Glu-D1* genomic region and the origin of *Glu-D1d*. Using seven diagnostic DNA markers, 12 *Glu-D1* haplotypes were detected among common wheat, European spelt wheat (*T. spelta*, a primitive hexaploid relative of common wheat), and *Aegilops tauschii* (the D genome donor of hexaploid wheat). By comparatively analyzing *Glu-D1* haplotypes and their associated *1Dx* and *1Dy* genes, we deduce that the haplotype carrying *Glu-D1d* was likely differentiated in the ancestral hexaploid wheat around 10,000 years ago, and was subsequently transmitted to domesticated common wheat and *T. spelta*. A group of relatively ancient *Glu-D1* haplotypes was discovered in *Ae. tauschii*, which may serve for the evolution of other haplotypes. Moreover, a number of new *Glu-D1d* variants were found in *T. spelta*. The main steps in *Glu-D1d* differentiation are proposed. The implications of our work for enhancing the utility of *Glu-D1d* in wheat quality improvement and studying the SSP alleles in other crop species are discussed.

## Introduction

Seed storage proteins (SSPs) occur in diverse plant species. They provide energy for seed germination and seedling growth, and form a major source of dietary protein for mankind [1]. In crop plants, SSPs are frequently crucial determinants of quality traits [1], [2]. Many superior SSP alleles have been identified and used in breeding (e.g., [3]–[5]), and substantial efforts have been devoted in sequencing the genomic regions containing SSP coding genes (e.g., [6]–[8]). In general, SSPs tend to be expressed from complex gene families, and often possess multiple alleles with different phenotypic effects [9], [10]. In order to more effectively use SSPs in improving crop quality traits, it is necessary to understand the variations of the genomic loci harbouring SSP genes in germplasm resources and the origins of superior SSP alleles.

High-molecular-weight glutenin subunits (HMW-GSs) are a family of SSPs essential for the end-use quality control of common wheat (*Triticum aestivum*, AABBDD) [4], [11]. HMW-GSs, together with the low-molecular-weight glutenin subunits (LMW-GSs) and gliadins, are the major factors conferring viscoelastic properties to wheat doughs. The relative balance of elasticity (controlled mainly by HMW-GSs and LMW-GSs) and viscosity (largely by gliadins) in the doughs determines their suitability for being processed into different types of wheat foods. In hexaploid wheat, the genes encoding HMW-GSs are contained in homoeologous *Glu-1* loci (*Glu-A1*, *B1*, and *D1*), which are located on the 1A, 1B and 1D chromosomes, respectively [11]. The loci orthologous to *Glu-A1*, *B1*, and *D1* have been found in the *Triticeae* species closely related to common wheat [12]. The genomic regions of *Glu-1* loci are structurally complex, with the core of a *Glu-1* locus being composed of four genes arranged in the order of *Globulin 1*−*Glu-1-2*−*Globulin 2*−*Glu-1-1* [13]. *Glu-1-1* and *Glu01-2* encode x- and y-type subunits, respectively. Owing to allelic variation and gene inactivation, both *Glu-1-1* and *Glu-1-2* have multiple alleles [14]. For example, *Glu-D1* has several commonly found alleles, *Glu-D1a*, *b*, *c*, *d*, *e* and *f* [4], [15]. The two HMW-GS genes of *Glu-D1a* are *1Dx2* and *1Dy12* (encoding 1Dx2 and 1Dy12 subunits, respectively), whereas those of *Glu-D1d* are *1Dx5* and *1Dy10* (specifying 1Dx5 and 1Dy10 subunits, respectively) [15], [16].

The primary structure of HMW-GSs is composed of a signal peptide (removed from mature subunit), a N terminal domain, a central repetitive domain, and a C-terminal domain [9]. The cysteine residues in HMW-GSs are usually conserved in both number and position. However, compared to 1Dx2, 1Dx5 harbors the amino acid substitution at position 118, causing the replacement of a serine residue by cysteine at the beginning of the repetitive domain [17]. Relative to 1Dx2, the presence of this extra cysteine in 1Dx5 is frequently associated with the doughs exhibiting stronger elasticity and superior end-use qualities for breadmaking and noodle processing [17]–[21]. Interestingly, several studies have also shown that the wheat varieties carrying *Glu-D1d* are generally more tolerant to heat stress-induced decline of dough quality than those having *Glu-D1a* [19], [20], [22], [23]. This suggests that *Glu-D1d* is likely to be even more useful for future improvement of wheat end-use qualities, as heat stress may become increasingly frequent in the next 30–50 years owing to global warming [24].

Despite its agronomical importance, the evolutionary origin of *Glu-D1d* remains a mystery. Since extant common wheat was descended from domestication of the ancestral hexaploid wheat formed about 10,000 years ago through natural hybridization between tetraploid wheat (*T. turgidum*, AABB) and the D genome donor *Aegilops tauschii* (DD) [25], [26], it is possible that *Glu-D1d* may come directly from *Ae. tauschii*, or be differentiated during hexaploidization. The *Glu-D1* genomic region has been sequenced in one *Ae. tauschii* accession and one common wheat variety [7], [27]. The *1Dx* and *1Dy* genes in the sequenced *Glu-D1* region are usually separated by more than 50 kb with each surrounded by multiple repetitive elements [7]. Furthermore, complex differences were found in not only *1Dx* and *1Dy* genes but also the repetitive elements between the *Glu-D1* region in *Ae. tauschii* and that of common wheat, indicating close relationships between the molecular variations of *1Dx* and *1Dy* genes and those of surrounding repetitive elements. Therefore, the main objectives of this work were to obtain a more comprehensive understanding of the haplotype variation of *Glu-D1* genomic region, and to shed new light on the origin of *Glu-D1d.* European spelt wheat (*T. spelta* thereafter), cultivated as land races, was included in this work because it possessed a hexaploid genome highly similar to that of common wheat, and represents a more primitive hexaploid relative of common wheat [28]–[31].

## Materials and Methods

### Plant Materials

**Figure 1:**
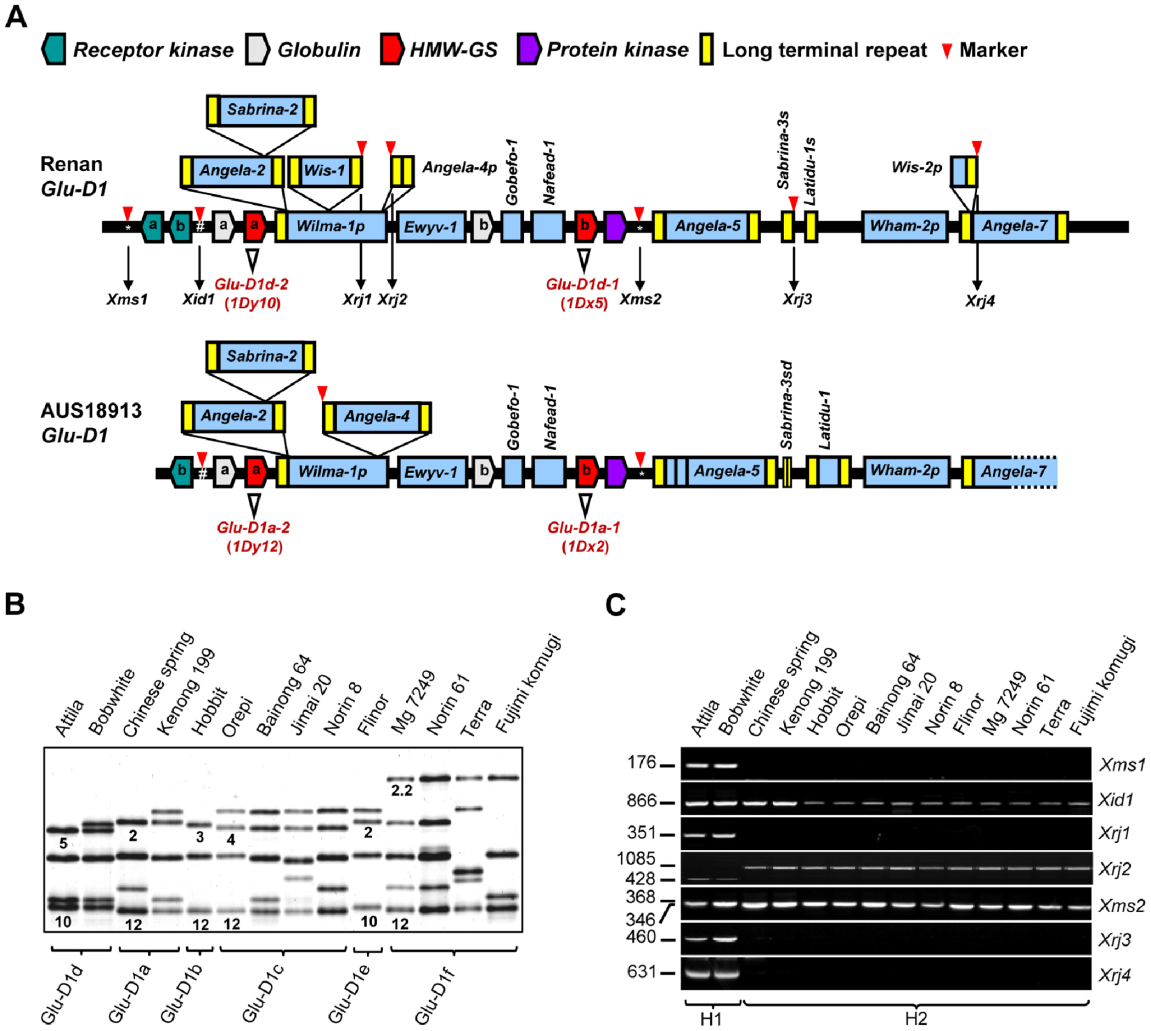
Development of seven new molecular markers in *Glu-D1* region. (A) Organization of homologous *Glu-D1* regions in the common wheat variety Renan and the *Ae. tauschii* accession AUS18913. The diagrams illustrating the organization of the various transposon elements and genes in the two *Glu-D1* regions were adapted from previously published works [7], [27]. Only the structures relevant to this work are shown. The letters “a” and “b” denote distinct copies of the duplicated genes. Positions of the seven newly developed DNA markers (*Xms1*, *Xid1*, *Xrj1*, *Xrj2*, *Xms2*, *Xrj3*, and *Xrj4*) are indicated by filled arrowheads. The “*” and “#” symbols indicate the markers based on microsatellite or indel. The *HMW-GS* genes *Glu-D1d-1* (*1Dx5*), *Glu-D1d-2* (*1Dy10*), *Glu-D1a-1* (*1Dx2*) and *Glu-D1a-2* (*1Dy12*) are marked by empty arrowheads. The *Wis-1* and *Wis-2p* insertions were unique to Renan *Glu-D1*. The *Angela-4* element, although intact in AUS18913 *Glu-D1*, had its internal region deleted in Renan *Glu-D1* (and thus named as *Angela-4p*). Compared to the solo-LTR *Sabrina-3s* in Renan *Glu-D1*, its counterpart in AUS18913 *Glu-D1* underwent further sequence deletion (and thus designated as *Sabrina-3sd*). (B) SDS-PAGE analysis of HMW-GS subunits from 14 common varieties containing *Glu-D1a*, *b*, *c*, *d*, *e* or *f* alleles. The 1Dx and 1Dy subunit pairs (1Dx5+1Dy10, 1Dx2+1Dy12, 1Dx3+1Dy12, 1Dx4+1Dy12, 1Dx2+1Dy10, 1Dx2.2+1Dy12) encoded by different *Glu-D1* alleles are indicated by Arabic numbers. (C) Amplification patterns of seven *Glu-D1* markers in the 14 common wheat lines with *Glu-D1a*, *b*, *c*, *d*, *e* or *f* alleles. The size (bp) of the amplicons and the names of the markers are provided on the left and right sides of the graph, respectively.

A total of 208 common wheat varieties (listed in Figure 1 and Tables S1 and S2), along with 355 *Ae. tauschii* accessions (Table S3) and 215 *T. spelta* lines (Table S4), were employed in this study to investigate *Glu-D1* haplotypes. Additional materials and the main suppliers of the germplasm stocks used in this work are provided in Methods S1.

### Marker Development and Haplotype Analysis

The BAC sequences of DQ537337 (from Renan) and AF497474 (from *Ae. tauschii* accession AUS18913) harboring *Glu-D1* locus were retrieved from NCBI database (http://www.ncbi.nlm.nih.gov/). Three types of polymorphisms, including transposable element (TE) insertions or deletions, simple sequence repeats, and short nucleotide indels, between the two sequences were considered for marker development. The software Primer Premier 5.0 (PREMIER Biosoft International, CA, USA) was used for designing the primer pairs specific for each of the seven *Glu-D1* markers (Table S5). Genomic DNA samples were extracted from desired plant lines grown in the greenhouse using a CTAB method [32], and used for PCR amplifications (Methods S1). *Glu-D1* haplotypes were distinguished by differences in the alleles of the seven diagnostic markers.

### Detection of *1Dx* and *1Dy* Genes using the PCR Markers UMN25 and UMN26

The PCR markers UMN25 and UMN26 developed by Liu *et al.* [33] were used to detect the *1Dx* and *1Dy* genes in one set of common wheat lines (Tables S2), and to investigate the *1Dy* genes hosted by 12 *Glu-D1* haplotypes. PCR amplification and gel analysis were conducted as described previously [33].

### Isolation of *1Dx* or *1Dy* Coding Sequences and the Long Terminal Repeat (LTR) Regions of *Sabrina-2*

The primers for amplifying *1Dx* or *1Dy* coding sequences and the left or right LTR regions of *Sabrina-2* (Table S5) were developed using Primer Premier 5.0 (PREMIER Biosoft International, CA, USA). Genomic PCR was employed for isolating the desired sequences, which was always conducted using the high fidelity DNA polymerase ExTaq (TaKaRa, Tokyo, Japan), with the cycling parameters identical to those reported previously [34]. The PCR fragments of the expected size were cloned and sequenced (Methods S1). To facilitate recognition, the *1Dx* (or *1Dy*) genes from *T. spelta* and *Ae. tauschii* were labeled by *1D^s^x* (*1D^s^y*) and *1D^t^x* (*1D^s^y*), respectively. Additionally, each of the newly characterized *1Dx* (*1D_y_*) gene was tagged by the name and *Glu-D1* haplotype of the accession from which it was isolated.

### Dating LTR Retrotransposon Insertion Times

The insertion time of retroelement was estimated based on nucleotide substitution rate in the left and right LTR sequences according to a previous study [35]. Kimura-2 parameter distances (K) between the two LTRs of individual elements were calculated by software MEGA 5.0 [36]. The average substitution rate (r) of 1.3×10^−8^ substitutions per synonymous site per year [37], was used to date the insertions of retroelements. The time (*T*) since element insertion was calculated using the formula *T* = *K/*2r [38].

### Multiple Alignment and Phylogenetic Analysis

Sequence alignment was performed using the ClustalW program (www.ebi.ac.uk). The resultant multiple alignment was then used for constructing phylogenetic trees using the software MEGA 5.0 (Methods S1).

### Estimation of Approximate Divergence Times of *1Dx* or *1Dy* Genes

Synonymous substitution (Ks) was estimated with MEGA 5.0 [36], Divergence time (*T*) was calculated using *T* = *K*s/2*r*, where *r* is the grass *Adh1* and *Adh2* substitution rate of 6.5×10^−9^ substitutions per synonymous site per year [39].

### Phylogenetic Network Analysis

The alleles of the seven *Glu-D1* markers and the types of the *1Dx* and *1Dy* genes were employed for phylogenetic network analysis of 12 *Glu-D1* haplotypes with the Median-Joining network algorithm [40], which is installed in the software network 4.6.1.0 (Fluxus Technology Ltd., Suffolk, UK).

### Accession Numbers

All novel sequences were submitted to GenBank with reference numbers JX173931–JX173953.

## Results

### Development of New DNA Markers for *Glu-D1* Locus

The genomic sequences available for *Glu-D1* regions in the *Ae. tauschii* accession AUS18913 and common wheat variety Renan allowed for understanding structural variations in this locus between the diploid D and hexaploid D genomes [7], [27]. The *Glu-D1* allele of Renan encodes 1Dx5 and 1Dy10 [41], and thus belongs to *Glu-D1d*, whereas the *Glu-D1* allele of AUS18913 is likely *Glu-D1a*, because the two HMW-GSs in this accession resemble highly 1Dx2 and 1Dy12 [27]. The patterns of transposon insertion and deletion vary considerably between the two *Glu-D1* regions (Figure 1A and [7]). We therefore developed seven new DNA markers based on their sequence variations to investigate the haplotype structure of *Glu-D1* locus (Figure 1A). Among these markers, two (*Xms1* and *Xid1*) were located upstream of *1Dy* (*Glu-D1–2*), two (*Xrj1* and *Xrj2*) resided between *1Dy* and *1Dx* (*Glu-D1-1*), and the remaining three (*Xms2*, *Xrj3* and *Xrj4*) were downstream of *1Dx* (Figure 1A). *Xms1* and *2* were two microsatellite markers, and *Xid1* was an indel marker. *Xrj1*, *2*, *3* and *4* were repeat DNA insertion site based polymorphism (ISBP) markers [42], [43]. *Xrj1* and *2* were designed based on the unique insertion of the retrotransposon *Wis-1* and specific deletion of the internal region in the retroelement *Angela-4* in Renan *Glu-D1* (Figure 1A). *Xrj3* was developed based on structural difference between *Sabrina-3s* and *Sabrina-3sd* in Renan and AUS18913 *Glu-D1* regions, while *Xrj4* was derived from the specific presence of another partially deleted retrotransposon (*Wis-2p*) in Renan *Glu-D1* (Figure 1A). The location of the seven markers in the 1D chromosome was confirmed by PCR mapping (Figure S1).

The capacity of the newly developed markers for revealing potential haplotype variation of *Glu-D1* was tested by examining 14 common wheat varieties carrying *Glu-D1a*, *b*, *c*, *d*, *e* or *f* alleles (Figure 1B). Clearly, two distinct haplotypes (H1 and H2) were detected, with H1 unique to the two varieties (Attila and Bobwhite) carrying *Glu-D1d* and H2 shared by the remaining 12 varieties harboring *Glu-D1a*, *b*, *c*, *e* or *f* (Figure 1C). H1 was characterized by positive amplifications of all seven markers. In contrast, H2 was characterized by positive amplifications of *Xid1*, *Xrj2* and *Xms2*, but not *Xms1* or *Xrj1*, *3* and *4*. Moreover, the alleles of *Xid1*, *Xrj2* or *Xms2* amplified for the varieties carrying *Glu-D1a*, *b*, *c*, *e* or *f* were identical, indicating that the three markers were monomorphic, and could not differentiate, among the five *Glu-D1* alleles. Among the three markers co-dominant for H1 and H2, the amplicon size of *Xrj2* was smaller in H1 than in H2 (Figure 1C), which was due to sequence deletion in *Angela-4p* in the *Glu-D1* region represented by Renan (Figure 1A). The amplicon lengths of *Xms2* in H1 and H2 were 368 and 346 bp, respectively (Figure S2), which corresponded to a 22 bp indel between the two amplicons of this microsatellite marker in Renan and AUS18913. Finally, the amplicon size of *Xid1* did not differ between the two *Glu-D1* haplotypes detected in the 14 common wheat genotypes.

### Detection and Analysis of *Glu-D1* Haplotypes

The haplotype variation of *Glu-D1* locus in common wheat was further investigated using two larger sets of materials. The first set was composed of 60 common wheat varieties from diverse geographic locations and with known information on their *Glu-D1* alleles (Table S1). After genotyping with the seven diagnostic *Glu-D1* markers, H1 was specifically detected in the varieties carrying *Glu-D1d*, whereas H2 was shared by the remaining having other *Glu-D1* alleles (Table S1). In the second set of 134 common wheat varieties, the status of their *Glu-D1* alleles was unknown before this work. After screening by PCR amplification of the co-dominant markers UMN25 (co-dominant for *1Dx2* and *1Dx5*) and UMN26 (co-dominant for *1Dy12* and *1Dy10*) [33], these lines were divided into two groups, one carrying *Glu-D1d* and the other with *Glu-D1a*. Upon genotyping with the seven *Glu-D1* markers, the varieties having *Glu-D1d* were all found to belong to H1, while those harboring *Glu-D1a* alleles could all be assigned to H2 (Table S2). Clearly, H1 and H2 were the two main haplotypes of *Glu-D1* locus in common wheat. H1 was specific for *Glu-D1d*, whereas H2 was shared by *Glu-D1a*, *b*, *c*, *e* or *f*.

Using the seven markers, a total of nine *Glu-D1* haplotypes was identified among 355 diverse *Ae. tauschii* accessions collected from over 20 countries (Table 1, Table S3). Compared with common wheat, *Xid1* detected additional polymorphisms in *Ae. tauschii*, but *Xrj2* and *Xrj4* were both monomorphic (Table 1). Among the nine haplotypes found in *Ae. tauschii*, H3 was most common (found in 49.3% accessions), followed by H2 (in 23.7% accessions). The only difference between H2 and H3 was the positive amplification of *Xrj3* in the latter (Table 1). H4 was the third most frequent haplotype (in 9.6% accessions), and differed from H3 in the size of the product of *Xms2*, which was 368 bp in H4, but 346 bp in H3 (Table 1). Collectively, H2, H3 and H4 were found in 82.6% of the *Ae. tauschii* accessions examined. The remaining six haplotypes were detected in only small numbers of accessions (Table 1). The marker alleles of *Ae. tauschii* H2 were identical to those of common wheat H2 (Table 1). Surprisingly, we did not find H1 in the large number of *Ae. tauschii* accessions analyzed in this work.

**Table 1:** *Glu-D1* locus haplotypes detected in *T. aestivum*, *T. spelta* and *Ae. tauschii* populations.

Haplotype	Marker							Species
	*Xms1*	*Xid1*	*Xrj1*	*Xrj2*	*Xms2*	*Xrj3*	*Xrj4*	
H1	176^a^	866	351	428	368	460	631	*T. spelta* (9, 4.2%)^c^ *T. aestivum* (99, 47.7%)
H2	–b	866	–	1085	346	–	–	*Ae. tauschii* (84, 23.7%)*T. spelta* (175, 81.4%)*T. aestivum* (109, 52.3%)
H3	–	866	–	1085	346	460	–	*Ae. tauschii* (175, 49.3%)
H4	–	866	–	1085	368	460	–	*Ae. tauschii* (34, 9.6%)
H5	176	866	–	1085	368	460	–	*Ae. tauschii* (3, 0.8%)
H6	176	866	–	1085	346	–	–	*Ae. tauschii* (10, 2.8%)
H7	176	663	–	1085	346	–	–	*Ae. tauschii* (20, 5.6%)
H8	176	663	351	1085	346	–	–	*Ae. tauschii* (4, 1.1%)
H9	–	866	351	1085	346	–	–	*Ae. tauschii* (6, 1.7%)
H10	176	866	351	1085	346	–	–	*Ae. tauschii* (19, 5.4%)
H11	–	866	–	1085	346	–	631	*T. spelta* (30, 14.1%)
H12	176	866	351	1085	368	460	631	*T. spelta* (1, 0.5%)

a Length (bp) of amplified fragment by the corresponding marker.

b Null allele.

c Items in the brackets indicate the number and percentage of lines in which the given *Glu-D1* haplotype was detected.

A total of 215 *T. spelta* accessions (Table S4) was genotyped with the seven *Glu-D1* markers, resulting in the identification of four haplotypes (H1, H2, H11, and H12, Table 1). H1, although not found in *Ae. tauschii*, was detected in *T. spelta*. On the other hand, H11 and H12 were found in *T. spelta*, but not *Ae. tauschii* or common wheat. H2 was the predominant *Glu-D1* haplotype, followed by H11. H11 differed from H2 in the positive amplification of *Xrj4*, and H12 differed from H1 in the size of the product of *Xrj2*, which was 428 bp in H1, but 1085 bp in H12 (Table 1).

All together, 12 unique haplotypes were identified for *Glu-D1* locus in the *T. aestivum*, *Ae. tauschii*, and *T. spelta* materials in this study (Table 1). An important feature shared by H1, H11, and H12 was the positive amplification of *Xrj4*, which was not the case for the nine *Glu-D1* haplotypes in *Ae. tauschii*. Among the 12 haplotypes, only H1 had the 428 bp allele at *Xrj2* (Table 1).

### Approximate Timing for the Differentiation of *Glu-D1* Haplotypes

To estimate the time periods in which the different *Glu-D1* haplotypes were differentiated, transposon insertion and deletion events found in subsets of the 12 haplotypes were investigated, followed by molecular clock analysis using nucleotide substitutions in the LTRs of retroelements. Based on structural differences between the *Glu-D1* locus sequences of Renan and AUS18913 (Figure 1A), and the polymorphic amplification patterns of *Xrj1*, *2* and *3* (Table 1), the 12 haplotypes could be divided into five categories (Table 2). The H2 to H7 haplotypes in category I all contained the insertion of an intact *Angela-4* element, but lacked those by *Wis-1*, *Wis-2p* and *Angela-4p*. Molecular dating with *Angela-4* indicated that the insertion of this element into *Glu-D1* region took place approximately 1.42 MYA (Table 2), suggesting that the six haplotypes in category I were differentiated around 1.42 MYA. H8 to H10 in category II were characterized by possessing both *Angela-4* and *Wis-1* (Table 2). The insertion of *Wis-1* into *Glu-D1* region was estimated to be approximately 0.46 MYA based on nucleotide substitutions in the two LTRs of this element (Table 2). Therefore, H8 to H10 arose possibly around 0.46 MYA. The haplotypes in category III (H11), IV (H12) or V (H1) all contained the *Wis-2p* element with only one LTR. Therefore, the insertion time of *Wis-2p* could not be dated. However, considering that the *Glu-D1* haplotypes harboring *Wis-2p* were found in only common wheat (H1) and *T. spelta* (H1, H11 and H12), but not in *Ae. tauschii* (Table 1), *Wis-2p* insertion might occur after the hexaploidization event that gave rise to the ancestral hexaploid wheat (around 0.01 MYA). Thus, the *Glu-D1* haplotypes in categories III, IV or V might all be differentiated relatively recently compared to those in categories I and II, possibly around 0.01 MYA (Table 2).

**Table 2:** Approximate differentiation times of five categories of *Glu-D1* haplotypes.

TE event	Haplotype	TE used forestimation	Rough timing ofdifferentiation (MYA)	Species
+*Angela-4* a- *Wis-1*- *Wis-2p*-*Angela-4p*	H2 to H7	*Angela-4*	1.42±0.18	*Ae. tauschii* (H2 to H7)*T. spelta* (H2)*T. aestivum* (H2)
+*Angela-4*+*Wis-1*- *Wis-2p*-*Angela-4p*	H8 to H10	*Wis-1*	0.46±0.09	*Ae. tauschii*
+*Angela-4*- *Wis-1*+*Wis-2p*-*Angela-4p*	H11	*Wis-2p*	0.01	*T. spelta*
+*Angela-4*+*Wis-1*+*Wis-2p*-*Angela-4p*	H12	*Wis-2p*	0.01	*T. spelta*
− *Angela-4*+*Wis-1*+*Wis-* *2p*+*Angela-4p*	H1	*Wis-2p*	0.01	*T. speltaT. aestivum*

a The plus and minus symbols indicate the presence or absence of TE insertion.

The differentiation of H1 and H12 occurred around similar times was further investigated by analyzing the terminal sequences of the LTR element *Sabrina-2*, which existed in the *Glu-D1* region of both Renan and AUS18913 (Figure 1A). This element was inserted into *Glu-D1* locus around 2.01–2.30 MYA based on molecular dating using its LTR sequences (Table S6). No nucleotide substitution was found between the LTR sequences of the *Sabrina-2* elements resided in H1 and H12, although such substitutions existed between the *Sabrina-2* elements hosted by H1 and H5 or by H1 and H10 (Table S7). Further examination revealed that the number of nucleotide substitutions in *Sabrina-2* LTR sequences between H1 and H5 was substantially higher than that between H1 and H10 (Table S7). This observation was consistent with the findings that H5 was differentiated earlier than H10, and H10 was formed earlier than H1 (Table 2).

### Characterization of *1Dx* Genes and their Deduced Proteins

Because a major difference between *Glu-D1d* and *Glu-D1a* lies in their 1Dx subunits, we conducted sequence analysis of the *1Dx* genes in a range of *Ae. tauschii* and *T. spelta* accessions with their *Glu-D1* locus haplotyped in this study. A total of 20 unique *1Dx* ORFs was analyzed at both coding sequence and deduced protein levels by comparing with the two well characterized common wheat *1Dx* genes, *1Dx5* and *1Dx2* that belonged to *Glu-D1d* or *Glu-D1a* and were carried by *Glu-D1* haplotypes H1 and H2, respectively. The 20 *1Dx* ORFs were all intact and active because their products could be found in the grains by SDS-PAGE analysis of seed protein extracts (Figure S3).

Like the coding sequences of *1Dx5* and *1Dx2*, the newly cloned *1Dx* ORFs contained no intron. The 20 deduced 1Dx proteins from these accessions shared a highly similar primary structure consisting of a signal peptide (21 residues), a N-terminal domain (89 residues), a central repetitive domain (681 to 702 residues), and a C-terminal domain (42 or 43 residues) (Table 3). Their central repetitive domains were composed of mainly tandem and interspersed repeats with base units of tripeptide, hexapeptide, and nonapeptide motifs (Figure S4). For eight 1Dx subunits, the number of conserved cysteine residues and their relative positions in the protein sequence were identical to those in 1Dx5 (Table 3, Figure S4). For the remaining 12 1Dx subunits, the number of conserved cysteine residues and their relative positions in the protein sequence were the same as those in 1Dx2 (Table 3, Figure S4). Interestingly, the eight accessions with 1Dx5-like subunits all belonged to *T. spelta*, and their *Glu-D1* haplotype was H1; for the 12 accessions with 1Dx2-like subunits, nine belonged to *Ae. tauschii* and three to *T. spelta* (Table 3). Subsequently, we compared the substitution and indel patterns among 1Dx5, 1Dx2, and the 20 1Dx subunits. A total of 15 polymorphic sites, including 10 substitutions (S1 to S10) and five indels (ID1 to ID5), were analyzed. As shown in Table S8, each of the ten substitutions occurred between two residues. The S1 substitution, S118C, was linked to functional difference between 1Dx2 and 1Dx5 (see Introduction). The indels occurred at the five sites caused removal or addition of short peptides (Table S8). In general, the 1Dx subunits specified by *Glu-D1* haplotypes H3 to H5 showed the highest dissimilarities from both 1Dx5 and 1Dx2 in the 15 sites (Table S8). Remarkably, the 1Dx2-like subunit 1D^s^x-TRI19057^H12^, although lacking S118C substitution, strongly resembled 1Dx5 and 1Dx5-like subunits in the majority of the remaining sites; three 1Dx2-like subunits (1D^t^x-PI511368^H2^, 1D^s^x-PI348360^H2^ and 1D^s^x-TRI5008^H11^) were either highly similar, or identical to, 1Dx2 in these 15 sites (Table S8). Apart from the substitutions and indels described above, there also existed additional changes that were either specific to individuals, or shared by subsets, of the 20 compared 1Dx protein sequences (Figure S4). For example, extra and unique cysteine residues were found in the repetitive domain of the 1Dx5-like subunits 1D^s^x-TRI9883^H1^ and 1D^s^x-TRI16607^H1^ and the 1Dx2-like subunit 1D^s^x-TRI5008^H11^.

**Table 3:** Main features of the 20 newly isolated 1Dx subunits.

1Dx	ORF size (bp)	Deduced protein (aa)				
		SP	ND	RD	CD	Total
1Dx5	2550	21	89 (3)^a^	696 (1)^a^	42 (1)^a^	848 (5)^b^
1Dx2	2523	21	89 (3)	687 (0)	42 (1)	839 (4)
**1D^s^x-PI15865^H1^**	2550	21	89 (3)	696 (1)	42 (1)	848 (5)
**1D^s^x-PI361813^H1^**	2550	21	89 (3)	696 (1)	42 (1)	848 (5)
**1D^s^x-TRI9883^H1^**	2550	21	89 (3)	696 (2)	42 (1)	848 (6)
**1D^s^x-TRI16607^H1^**	2550	21	89 (3)	696 (2)	42 (1)	848 (6)
**1D^s^x-TRI16981^H1^**	2550	21	89 (3)	696 (1)	42 (1)	848 (5)
**1D^s^x-PI347904^H1^**	2550	21	89 (3)	696 (1)	42 (1)	848 (5)
**1D^s^x-PI348150^H1^**	2550	21	89 (3)	696 (1)	42 (1)	848 (5)
**1D^s^x-PI348171^H1^**	2550	21	89 (3)	696 (1)	42 (1)	848 (5)
1D^s^x-PI348360^H2^	2523	21	89 (3)	687 (0)	42 (1)	839 (4)
1D^t^x-PI511368^H2^	2532	21	89 (3)	690 (0)	42 (1)	842 (4)
1D^t^x-IG46663^H3^	2541	21	89 (3)	693 (0)	42 (1)	845 (4)
1D^t^x-PI603224^H4^	2541	21	89 (3)	693 (0)	42 (1)	845 (4)
1D^t^x-IG48561^H5^	2541	21	89 (3)	693 (0)	42 (1)	845 (4)
1D^t^x-PI603236^H6^	2505	21	89 (3)	681 (0)	42 (1)	833 (4)
1D^t^x-CIAE24^H7^	2541	21	89 (3)	693 (0)	42 (1)	845 (4)
1D^t^x-TA2527^H8^	2541	21	89 (3)	693 (0)	42 (1)	845 (4)
1D^t^x-PI349047^H9^	2568	21	89 (3)	702 (0)	43 (1)	855 (4)
1D^t^x-PI603223^H10^	2568	21	89 (3)	702 (0)	43 (1)	855 (4)
1D^s^x-TRI5008^H11^	2523	21	89 (3)	687 (1)	42 (1)	839 (5)
1D^s^x-TRI19057^H12^	2523	21	89 (3)	687 (0)	42 (1)	839 (4)

ORF: open reading frame; SP: signal peptide; ND: N-terminal domain; RD: repetitive domain; CD: C-terminal domain. The 1Dx5-like and 1Dx2-like subunits are shown in bold and underlined, respectively.

a Items in the brackets indicate the number of cysteine residues in the ND, RD or CD regions of each 1Dx protein.

b The number of total cysteine residues in each 1Dx subunit is provided in the brackets.

### Phylogenetic Analysis of *1Dx* Genes and Estimation of *1Dx* Divergence Times

**Figure 2:**
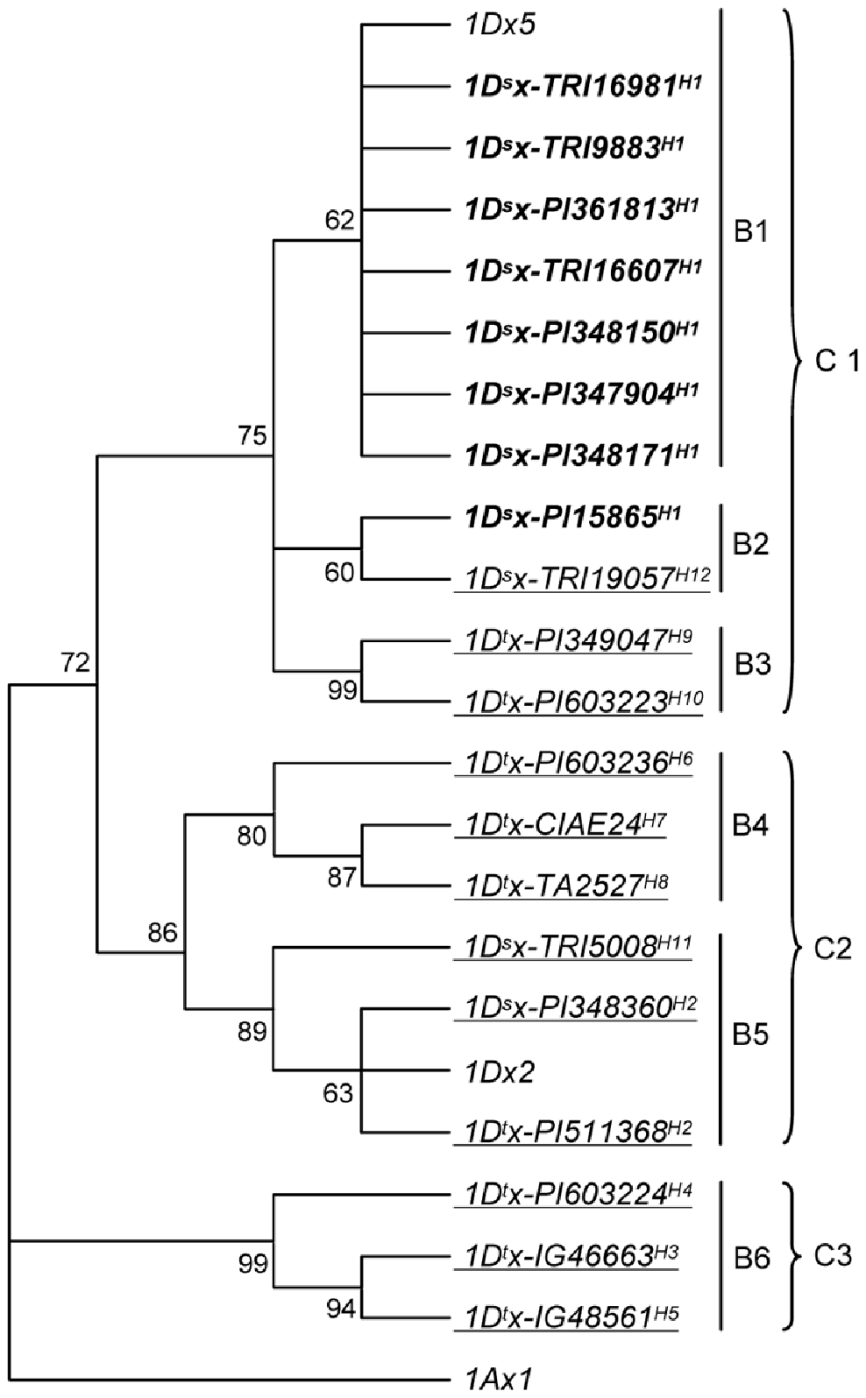
Phylogenetic analysis of *1Dx* genes. The tree shown was constructed using the multiple alignment of nucleotide sequences, and the neighbor joining program. Three distinctive clades (C1 to C3) and six major branches (B1 to B6) were observed. Highly similar trees were obtained with multiple alignment of deduced protein sequences, and an alternative tree building programs (i.e., minimum evolution). The *1Dx5*-like and *1Dx2*-like genes are shown in bold and underlined, respectively. *1Ax1* from common wheat *Glu-A1* locus was used as an outgroup control. Bootstrap values were obtained with 1000 permutations. The GenBank accession numbers for *1Dx5*, *1Dx2* and *1Ax1* are X12928, X03346 and X61009, respectively.

Phylogenetic analysis was conducted to investigate the relatedness among the 20 *1Dx* genes. In a typical neighbor joining tree constructed with full length nucleotide sequences (Figure 2), there were three well supported clades (C1 to C3), with C1 and C2 in the upper and parallel positions and C3 located at the basal side. Six major branches, including three (B1 to B3) in C1, two (B4 to B5) in C2, and one (B6) in C3 (Figure 2), could be recognized. Within C1, the top branch B1 was formed by *1Dx5* from common wheat and the seven *1Dx5*-like genes from *T. spelta*, the middle branch B2 was composed of the *1Dx2*-like gene *1D^s^x-TRI19057^H12^* and the *1Dx5*-like gene *1D^s^x-PI15865^H1^*, and the basal branch B3 contained two *1Dx2*-like genes from *Ae. tauschii* (Figure 2). Within C2, B4 was composed of exclusively *1Dx2*-like genes from *Ae. tauschii*, and B5 of *1Dx2* from common wheat and three *1Dx2*-like genes from *Ae. tauschii* and *T. spelta* (Figure 2). C3 contained the *1Dx2*-like genes from only *Ae. tauschii* (Figure 2). The *1Dx* genes aggregated in the three clades were derived from separate sets of *Glu-D1* haplotypes, namely, H1, H9, H10 and H12 for the genes in C1, H2, H6, H7, H8 and H11 for those in C2, and H3, H4 and H5 for those in C3 (Figure 2). Phylogenetic trees were also constructed using full length amino acid sequences and an alternative program (i.e., minimum evolution). The result was identical to that displayed in Figure 2.

On the basis of the phylogenetic analysis, the divergence times between the *1Dx* genes of hexaploid wheat and *Ae. tauschii* were estimated. In the C1 cluster, the averaged divergence time between *1Dx5* (representing hexaploid wheat *1Dx5* and *1Dx5-*like genes) and the two *Ae. tauschii 1Dx2*-like genes (*1D^t^x-PI349047^H9^* and *1D^t^x-PI603223^H10^*) was found to be around 0.29 MY (Table S9). In C2, the averaged divergence time between *1Dx2* (representing hexaploid wheat *1Dx2* and *1Dx2-*like genes) and the four *Ae. tauschii 1Dx2*-like genes (*1D^t^x-PI511368^H2^*, *1D^t^x-PI603236^H6^*, *1D^t^x-CIAE24^H7^* and *1D^t^x-TA2527^H8^*) was approximately 0.13 MY, with the specific time between *1Dx2* and *1D^t^x-PI511368^H2^* being only about 0.04 MYA (Table S9). Interestingly, the three *Ae. tauschii 1Dx2*-like genes in C3 exhibited relatively the longest averaged divergence times from both *1Dx5* (0.37 MY) and *1Dx2* (0.36 MY) (Table S9).

### Analysis of *1Dy* Genes and their Deduced Proteins

Initially, the *1Dy* genes hosted by different *Glu-D1* haplotypes were analyzed using the UMN26 marker that permits the distinction of *1Dy10* from *1Dy12* [33]. By comparing with the fragments amplified from the common wheat varieties Bobwhite (carrying *1Dy10*) and CS (harboring *1Dy12*), *1Dy10* (or *1Dy10*-like) was found in *T. spelta* haplotypes H1 and H12 and *Ae. tauschii* haplotypes H9 and H10, with *1Dy12* (or *1Dy12*-like) present in the remaining haplotypes (Figure S5). To verify the presence of intact *1Dy10* ORF, the *1Dy* coding sequence in the *Ae. tauschii* accession PI603223 (with haplotype H10) and the *T. spelta* accession TRI19057 (with haplotype H12) were cloned. To facilitate further comparisons, the *1Dy* coding sequence in the two *Ae. tauschii* accessions PI511368 and IG48561 (with haplotypes H2 and H5, respectively) and the *T. spelta* accession PI348360 (representing the H2 haplotype in spelt wheat) were also isolated. The expression of these *1Dy* genes was verified by SDS-PAGE analysis of seed protein extracts (Figure S3).

Like *1Dy10* and *1Dy12*, the five *1Dy* sequences (designated as *1D^t^y-PI603223^H10^*, *1D^t^y-PI511368^H2^*, *1D^t^y-IG48561^H5^, 1D^s^y-PI348360^H2^*, and *1D^s^y-TRI19057^H12^*, respectively) did not contain intron, and were all terminated by tandem stop codons. The primary structure of their deduced proteins was identical to that of 1Dy10 and 1Dy12 (Figure S6). Nucleotide sequence comparisons revealed that *1D^s^y-TRI19057^H12^* was identical to *1Dy10*, and *1D^t^y-PI603223^H10^* showed higher identity to *1Dy10* (99.6%) than to *1Dy12* (96.7%). In contrast, *1D^s^y-PI348360^H2^* was identical to *1Dy12*, and *1D^t^y-PI511368^H2^* exhibited higher identity to *1Dy12* (99.7%) than to *1Dy10* (96.7%). *1D^t^y-IG48561^H5^* displayed comparable identities to both *1Dy10* (96.2%) and *1Dy12* (97.9%).

### Phylogenetic Network Analysis of *Glu-D1* Haplotypes and a Possible Homologous Recombination Event between H5 and H10

**Figure 3:**
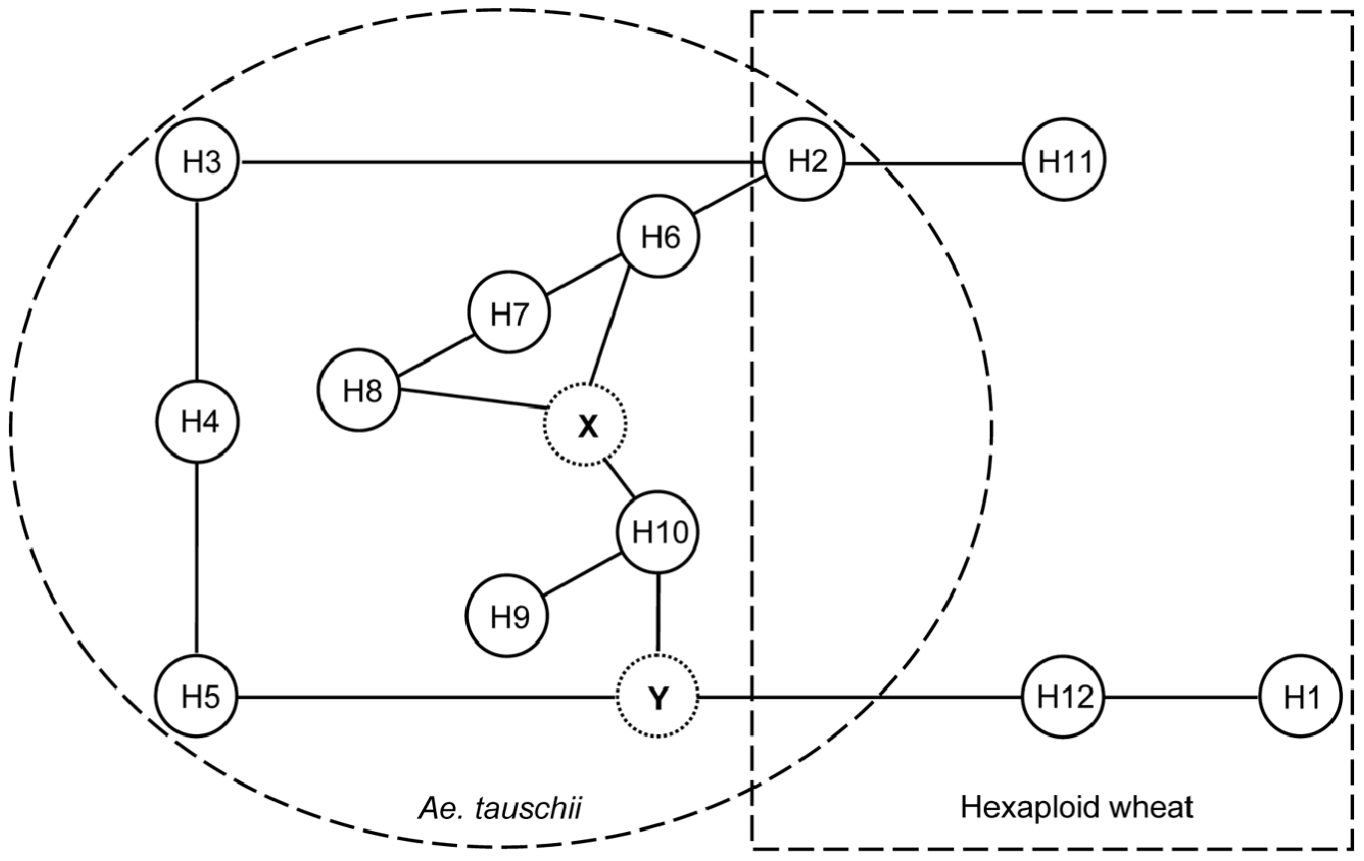
Phylogenetic network analysis of 12 *Glu-D1* haplotypes. The haplotypes detected in *Ae. tauschii* were circled, whereas those in *T. spelta* and common wheat were boxed. H2 was a shared haplotype. Two hypothetical intermediate haplotypes (X and Y) were predicted by the network analysis program.

Based on the variations of *1Dx* and *1Dy* genes and the similarities and differences in the alleles of the seven DNA markers (Figure S7), a phylogenetic network analysis was conducted to investigate relationships among the 12 haplotypes. In *Ae. tauschii*, direct network connection was found among H2, H3, H4, H5, H6, H7 and H8, as well as between H9 and H10 (Figure 3). In common wheat and *T. spelta*, direct connection was found between H12 and H1, and between H2 and H11 (Figure 3). Two hypothetical intermediate haplotypes were predicted by the analysis program, one (X) connecting H6, H7 and H8 to H9 and H10, and the other (Y) linking H5 and H10 to H12 (Figure 3).

**Figure 4:**
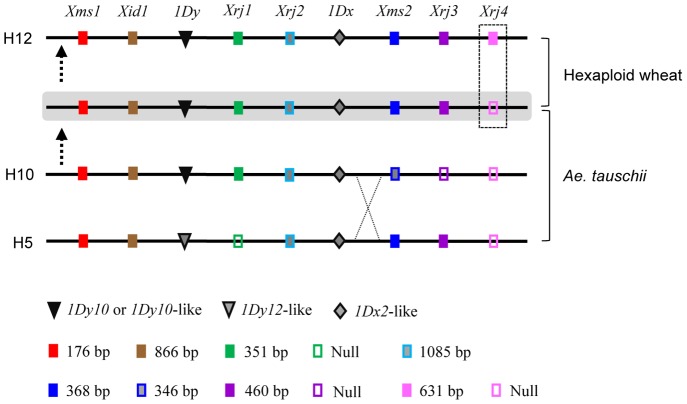
A possible homologous recombination event between *Glu-D1* haplotypes H5 and H10. This event might take place in *Ae. tauschii* between H5 and H10 downstream of the *1Dx* gene (as indicated by the cross). The descendent was introduced to the ancestral hexaploid wheat via hexaploidization, giving rise to H12 after further differentiation (as evidenced by the positive amplification of *Xrj4* due to *Wis-2p* insertion, boxed area). The types of *1Dx* and *1Dy* genes and the alleles of the seven *Glu-D1* markers hosted by the different haplotypes were indicated.

The linkage of both H5 and H10 to H12 was of particular interest considering the direct relationship between H12 and H1 that carries *Glu-D1d*. A simple explanation could be that both H5 and H10 might contribute to the differentiation of H12 through a recombination event. Judging from the alleles of *1Dx*, *1Dy* and the seven diagnostic markers, H12 resembled H10 in the upstream portion and including *Xrj2* (Figure 4). In the downstream portion of *Xrj2*, H12 shared two identical marker alleles (specified by *Xms2* and *Xrj3*, respectively) with H5, although the two haplotypes differed in the alleles of *1Dx* and *Xrj4* (Figure 4). We thus speculated that a homologous recombination event might have happened between H5 and H10 at a position downstream of *1Dx*, resulting in the prototype of H12 (Figure 4). In the prototype H12, the segment with the *1Dx* and *1Dy* genes was dissented from H10, whereas the remaining segment was derived from H5. Since neither H5 nor H10 were detected in extant common wheat and *T. spelta* (Table 1), the recombination event yielding the prototype H12 occurred in *Ae. tauschii*.

Consistent with the homologous recombination hypothesized above, it was found that *1D^t^x-PI603223^H10^* and *1D^t^y-PI603223^ H10^* showed higher identities to *1Dx5* and *1Dy10* (98.8% and 99.6%, respectively) than to *1Dx2* and *1Dy12* (96.2% and 96.7%, respectively). On the other hand, *1D^t^x-IG48561^H5^* and *1D^t^y-IG48561^H5^*, derived from the *Ae. tauschii* accession with haplotype H5, exhibited lower levels of identities to *1Dx5* and *1Dy10* (97.5% and 96.2%, respectively).

## Discussion

In this study, three complementary approaches were taken in order to investigate haplotype variation of *Glu-D1* and the origin of *Glu-D1d*. The development and application of seven diagnostic markers distributed in *Glu-D1* region permitted a rapid survey of the haplotype variation of this locus in several hundred germplasm lines. Molecular cloning of *1Dx* and *1Dy* coding sequences provided accurate gene sequence information in important *Glu-D1* haplotypes. Phylogenetic analysis and molecular dating were deployed to understand the relationships and divergence times of different *Glu-D1* haplotypes and the associated *1Dx* and *1Dy* genes. The new insights gained and their implications for further research are discussed below.

### Major Characteristics of *Glu-D1* Haplotypes

First, the number of *Glu-D1* haplotypes is highest in *Ae. tauschii* (totally nine), intermediate in *T. spelta* (four), and relatively low in common wheat (only two). Considering that more than two hundred diverse common wheat varieties had been haplotyped, the low number of *Glu-D1* haplotypes in common wheat is unlikely due to a biased selection of accessions. Since more than 200 *T. spelta* lines were genotyped, the four haplotypes revealed is likely a reasonable representation of the variation of *Glu-D1* region in this species.

Second, most *Glu-D1* haplotypes are not shared among *Ae. tauschii*, *T. spelta*, and *T. aestivum*, and haplotypes H1, H11 and H12 are not present in *Ae. tauschii*. Among the 12 haplotypes detected, only H2 was shared among common wheat, *T. spelta* and *Ae. tauschii*, and H1 by common wheat and *T. spelta*. H2 was the dominant haplotype in both common wheat and *T. spelta*, and is the second most abundant haplotype in *Ae. tauschii*. In contrast, H1 was not detected in *Ae. tauschii*, and a relatively rare haplotype (compared to H2 and H11) in *T. spelta*. The failure in detecting H1 in *Ae. tauschii* is consistent with the fact that typical 1Dx5 subunit with the S118C substitution has not been found in this species by this and previous studies [44], [45]. Therefore, it is highly probable that H1 (and the *Glu-D1d* allele contained therein) is absent in *Ae. tauschii*. A potential concern on this suggestion is that H1 may exist in *Ae. tauschii* in a very low frequency and was not detected by the present study. However, the failure of detecting H1 in 355 diverse *Ae. tauschii* accessions collected from over 20 countries (Table 1, Table S3), the presence of *Xrj2* and *Xrj4* in H1 but none of the *Ae. tauschii Glu-D1* haplotypes (Table 1, Figure S7), and the coincidence between the times of H1 differentiation and hexaploidization (Table 2) are strongly in favor of the emergence of H1 in hexaploid wheat but not *Ae. tauschii*. Although detected in *T. spelta*, H11 and H12 were also not found in *Ae. tauschii*. Because H11 and H12 shared the *Wis-2p* insertion with H1 (Table 1), and may be differentiated around the same time as H1, it is possible that, like H1, H11 and H12 are also absent in *Ae. tauschii*.

Third, some *Glu-D1* haplotypes are relatively ancient, whereas others may have emerged only recently. H2 to H10 were already present in *Ae. tauschii* before the hexaploidization event around 10,000 years ago. In contrast, H1, H11, and H12 may be emerged in more recent times (around 0.01 MYA, Table 2), coinciding with the time frame in the formation of the ancestral hexaploid wheat. Among H2 to H7 (differentiated around 1.42 MYA), H3 to H5 might be differentiated earlier, and H2, H6 and H7 might be derived from H3 to H5 (after further sequence deletion in *Sabrina-3s*). The direct derivation of H2 from H3 is also supported by a straight connection between the two haplotypes in the phylogenetic network analysis (Figure 3). On the other hand, H8 to H10 were differentiated later than H2 to H7 (around 0.46 MYA, Table 2).

Fourth, comparative analyses among *1Dx* or *1Dy* genes provide further clues on the differentiation of *Glu-D1* haplotypes. The *1Dx* alleles carried by H3 to H5 clustered together (Figure 2), and exhibited relatively the longest divergence times to both *1Dx5* and *1Dx2* (Table S9), supporting the idea that H3 to H5 may be the most ancient ones among the 12 haplotypes. The *1Dx* alleles from H1, H9, H10 and H12 aggregated together, with those from H9 and H10 at the basal position (Figure 2), pointing to the involvement of H9 and H10 in the evolution of H12 and H1. The *1Dx* alleles from H2, H6 to H8, and H11 formed another well supported cluster (Figure 2), with the *1Dx* allele from the *Ae. tauschii* H2 haplotype at the most basal position, indicating that the *Ae. tauschii* H2 is important for the evolution of H6 to H8, the presence of H2 in *T. spelta* and common wheat, and the emergence of H11 in *T. spelta*. The discovery of *1Dy10-*like gene in H10 and *1Dy10* in H12 further supports the role of the two haplotypes in the evolution of H1 that carries *1Dx5* and *1Dy10*. On the other hand, the finding of *1Dy12-*like gene in *Ae. tauschii* H2 and *1Dy12* in *T. spelta* H2 reinforces the likelihood that the H2 in *T. spelta* and common wheat was directly derived from the H2 of *Ae. tauschii* due to hexaploidization. Lastly, the direct relationship between *Ae. tauschii* H2 and the H2 in hexaploid wheat was also consistent with the very short divergence time between the *1Dx2*-like gene (e.g., *1D^t^x-PI511368^H2^*) in *Ae. tauschii* and *1Dx2* of common wheat (Table S9).

Finally, homologous recombination may contribute to the diversification of *Glu-D1* haplotypes in *Ae. tauschii*. In higher plants, haplotype variation and diversification of genes and chromosomal loci are usually caused by multiple means [46]. From the data presented in Figure 1 and Table 1, it is clear that haplotype variation of *Glu-D1* is caused by several reasons. These include nucleotide sequence alterations in two microsatellites (*Xms1* and *Xms2*), the indel in *Xid1*, and the mutations associated with four TEs (*Wis-1*, *Angela-4*, *Sabrina-3s*, and *Wis-2p*). Furthermore, we hypothesized that a homologous recombination event could have happened between H5 and H10 in *Ae. tauschii*, leading to further diversification of *Glu-D1* haplotypes (Figure 4). The involvement of homologous recombination in the differentiation of new haplotypes has also been reported in other plant species [47], [48].

### Important Steps in the Differentiation of H1 Haplotype Containing *Glu-D1d*

Given the discussion above, the network relationships among 12 *Glu-D1* haplotypes (Figure 3), and the hypothetical recombination between H5 and H10 (Figure 4), a simplified model could be drawn on possible derivative relationships among 12 *Glu-D1* haplotypes. In this model (Figure S8), H3, H4 and H5 are probably the founder haplotypes, H2 (derived from H3), H5 and H10 play an important role in the diversification of *Glu-D1* haplotypes. H2 is likely to give rise to a number of haplotypes (H6 to H10) in *Ae. tauschii*, and to H11 after being introduced into hexaploid wheat through hexaploidization.

Compared to H2 and H11, the route of H1 differentiation is more complex. Three steps may be important for the evolution of H1 containing *Glu-D1d* (Figure S8). The first one is the differentiation of H10, hosting a *1Dx2-*like allele and a *1Dy10-*like allele in *Ae. tauschii*. This step likely occurred around 0.46 MYA. The second step is the differentiation of H12 in the ancestral hexaploid wheat. This step might take place around 0.01 MYA. The source material for H12 differentiation might be the descendant from the homologous recombination event between H5 and H10, which was introduced to the ancestral hexaploid wheat via hexaploidization. The *1Dx2-*like allele in H12, although encoding a 1Dx subunit lacking the S118C substitution, was highly similar to *1Dx5* (Figure 2, Table S8). The *1Dy* gene in H12 was identical to *1Dy10*. The third step is the differentiation of H1, containing *Glu-D1d* allele and specifying the expression of 1Dx5 (or 1Dx5-like) and 1Dy10 subunits, from H12. This step might happen during or shortly after hexaploidization, with the resultant H1 retained in both common wheat and *T. spelta* populations.

### Impact of Hexaploidization and Domestication on *Glu-D1* Haplotypes

As a hexaploid crop species, the genetic structure of common wheat has been greatly influenced by hexaploidization, domestication, and breeding selection [31]. The strong effects of these processes on the birth and dissemination of new haplotypes are well demonstrated by recent studies on the powdery mildew resistance gene *Pm3* and the *Lr34* gene conferring multi-pathogen resistance [49]-[51]. A preponderance of the haplotype data obtained in this study leads us to suggest that the differentiation and dissemination of *Glu-D1* haplotypes might have also been affected by hexaploidization, domestication, and breeding in at least two aspects.

First, as noted above, the number of *Glu-D1* haplotypes detected varied substantially among *Ae. tauschii*, *T. spelta*, and common wheat. The most probable explanation for the low degree of haplotype diversity in common wheat may be genetic bottlenecks associated with hexaploidization (i.e., formation of the ancestral hexaploid wheat by hybridization between tetraploid wheat and *Ae. tauschii*) and domestication and breeding (from ancestral hexaploid wheat to extant common wheat varieties). It is well known that nucleotide diversity is substantially higher in *Ae. tauschii* genome than in the D genome of contemporary common wheat [52], and that relative few types (or individuals) of *Ae. tauschii* were involved in the formation of ancestral hexaploid wheat [53]–[55]. Moreover, it is widely accepted that human selection tends to further reduce the genetic diversity of plant and animal populations [31]. As land races, *T. spelta* accessions are less affected by artificial breeding efforts, and may have thus preserved more genetic diversities inherited from the ancestral hexaploid wheat [28], [29], [31]. This may, at least partly, be responsible for the existence of relatively more *Glu-D1* haplotypes in *T. spelta* than in common wheat. Several studies have speculated that *T. spelta* may be evolved from secondary hybridization between ancient free-threshing hexaploid wheat with hulled emmer wheat [28], [29], [56], [57]. This process might have also affected the differentiation and retention of *Glu-D1* haplotypes in *T. spelta*. Thus, the spectrum of *T. spelta Glu-D1* haplotypes revealed by this work might have been shaped by multiple forces (e.g., secondary hybridization, less artificial selection).

Second, the differentiation and dissemination of H1 (carrying *Glu-D1d* allele) might have been more intimately connected with hexaploidization and human selection. Terasawa et al. [58] found that the frequency of *Glu-D1d* was exceptionally high in the hexaploid wheat lines collected from Caucasian regions. Considering that common wheat originated near Caucasia [53], it is possible that H1 might be differentiated during or shortly after the hexaploidization event in Caucasia, and was subsequently selectively enriched in the free threshing common wheat populations around Caucasian regions due to the beneficial effects of *Glu-D1d* on end-use qualities [58]. As found in this work, H1 was a relatively rare haplotype compared to H2 and H11 in *T. spelta*, but its presence in extant common wheat almost equaled to that of H2 (Table 1). Because common wheat has been subjected to breeder’s selection more strongly than *T. spelta*, the increased presence of H1 in contemporary common wheat is largely caused by artificial breeding.

### Implications for Further Research

Apart from common wheat, there have also been substantial interests in recent years in identifying the elite alleles of SSPs in other crops, such as rice (e.g., [59], [60]), maize (e.g., [61]–[63]), and soybean (e.g., [5], [64]). As exemplified by this work, haplotype analysis with appropriate germplasm populations may be employed as an efficient strategy for increasing the understanding of SSP alleles, especially for those harbored by complex genomic loci and potentially impacted by genome polyploidization events. The resulting new knowledge may facilitate more effective use of SSP alleles in improving the quality traits of crops.

In this work, we found that the deduced protein sequences of the eight *T. spelta* 1Dx5-like subunits, although all carrying the S118C substitution (Table S8), differed considerably from that of the 1Dx5 subunit in common wheat (Figure S9). These *Glu-D1d* variants are likely valuable materials for further improvement of common wheat end-use qualities. We are now developing chromosomal introgression lines containing *T. spelta Glu-D1d* variants in common wheat background, which may be useful for testing the functionality of these new *Glu-D1d* variants. The seven DNA markers developed for *Glu-D1* region are being used for monitoring the transfer of *Glu-D1d* variants from *T. spelta* to common wheat. These markers may also be used in other end-use quality improvement programs involving the transfer of *Glu-D1d*, which are ongoing in many wheat production regions of the world [4], [65].

## Supporting Information

Figure S1 **Validation of the chromosomal specificity of the seven newly developed** ***Glu-D1*** **markers in this study.** Genomic DNA samples were extracted from Chinese Spring (CS), the nulli-tetrasomic line N1DT1A (lacking 1D chromosome and associated *Glu-D1* locus), the ditelosomic line of chromosomal arm 1DS (Dt1DS, lacking 1DL and associated *Glu-D1* locus), three additional common wheat genotypes (Attila, Bobwhite and Kenong 199), two tetraploid wheat genotypes (Langdon and Cofa), and two *Ae. tauschii* accessions (AUS18913 and AL8/78). They were then used in genomic PCR with the primer pairs for each of the seven markers (*Xms1*, *Xid1*, *Xrj1*, *Xrj2*, *Xms2*, *Xrj3*, and *Xrj4*). These markers did not amplify products in tetraploid wheat genotypes, N1DT1A, and Dt1DS, confirming that they are D genome specific, and that their target sequences are located on chromosomal 1DL on which *Glu-D1* resides. As anticipated, the seven markers showed polymorphisms in common wheat and between common wheat and *Ae. tauschii*. Lane M contains DNA size standard. The size (bp) of the amplified PCR fragments is shown on the left side of the graph. Items in the brackets indicate genome constitution of the examined wheat or *Ae. tauschii* genotypes.
(PDF)

Figure S2 **Multiple alignment of the DNA sequences of** ***Xms2*** **amplicons from Attila, Bobwhite, Chinese Spring (CS) and Kenong 199, as well as the reference sequences from Renan and AUS18913.** Chinese Spring (CS) and Kenong 199, as well as the reference sequences from Renan and AUS18913. The 22 bp deletion in the amplicons from CS, Kenong 199 and AUS18913 was indicated by dashed line.
(PDF)

Figure S3 **Detection of 1Dx and 1Dy subunits expressed by** ***T. spelta*** **and** ***Ae. tauschii Glu-D1*** **Haplotypes.** The 1Dx and 1Dy HMW-GSs in the two common wheat varieties, Bobwhite (expressing 1Dx5 and 1Dy10) and Chinese Spring (CS, expressing 1Dx2 and 1Dy12), and the *T. spelta* and *Ae. tauschii* accessions with different *Glu-D1* haplotypes were revealed by SDS-PAGE analysis of seed protein extracts. 1Dx and 1Dy subunits specified by different *T. spelta* and *Ae. tauschii Glu-D1* haplotypes were indicated by arrowheads and arrows, respectively. The protein band labeled by asterisk in H7 is a ω-gliadin protein.
(PDF)

Figure S4 **Multiple alignment of the deduced amino acid sequences of 1Dx5 and 1Dx2 subunits from common wheat, and the 20 1Dx subunits from** ***Ae. tauschii*** **and** ***T. spelta*** **.** The signal peptide is underlined, while the N- and C-terminal domains are labeled bold and bold italic, respectively. The repetitive domain is situated between the N- and C-terminal domains. The amino acid substitutions (S1 to S10) and indels (ID1 to ID5) between 1Dx5 and 1Dx2, and their variations in the 20 1Dx subunits from *T. spelta* and *Ae. tauschii*, are indicated. The four cysteine residues conserved among the 22 compared 1Dx subunits are marked by arrowheads. In addition, the cysteine reside unique to 1D^s^x-TRI9883^H1^, 1D^s^x-TRI16607^H1^ or 1D^s^x-TRI5008^H11^ is boxed.
(PDF)

Figure S5 **Detection of** ***1Dy*** **genes in the two common wheat varieties, Bobwhite (harboring** ***1Dy10*** **) and Chinese Spring (CS, containing** ***1Dy12*** **), and the** ***T. spelta*** **and** ***Ae. tauschii*** **accessions with different** ***Glu-D1*** **haplotypes using the PCR marker UMN26.** This marker is co-dominant for *1Dy10* and *1Dy12*, and allows the distinction between the two genes. Lane M contains DNA size standard (bp).
(PDF)

Figure S6 **Multiple alignment of the amino acid sequences of 1Dy10 and 1Dy12 subunits from common wheat and the five 1Dy subunits from** ***Ae. tauschii*** **and** ***T. spelta*** **.** The signal peptide is underlined, while the N- and C-terminal domains are labeled bold and bold italic, respectively. The repetitive domain resides between the N- and C-terminal domains. The amino acid substitutions (S1 to S12) and indels (ID1 to ID3) patterns between 1Dy10 and 1Dy12, and their variations in the five 1Dy subunits from *Ae. tauschii*, are indicated. The seven cysteine residues conserved among the seven compared 1Dy subunits are labeled by arrowheads.
(PDF)

Figure S7 **A diagram illustrating the variations of** ***1Dx*** **and** ***1Dy*** **genes and the alleles of the seven DNA markers in 12** ***Glu-D1*** **haplotypes.** The types of *1Dx* and *1Dy* genes and the alleles of the seven *Glu-D1* markers were indicated.
(PDF)

Figure S8 **A simplified model illustrating possible derivative relationships among 12** ***Glu-D1*** **haplotypes.** The arrows indicate the likely directions of haplotype differentiation. The approximate differentiation times (MYA) of the haplotypes in *Ae. tauschii* and hexaploid wheat were indicated. The three haplotypes (H5, H10 and H12) involved in the differentiation of H1 are labeled in bold. H2, detected in both *Ae. tauschii* and hexaploid wheat, was shown in italic.
(PDF)

Figure S9 **A diagram illustrating the positions of polymorphic sites among the 1Dx5 subunits from two common wheat varieties (Cheyenne and Renan) and the eight 1Dx5-like subunits from** ***T. spelta*** **genotypes.** 1Dx5 protein sequence (GenBank accession CAA31395) from Cheyenne was used as reference for locating the positions of polymorphic sites, where amino acid substitutions were detected. The diagram is not drawn in scale.
(PDF)

Table S1 **Haplotype variation of** ***Glu-D1*** **locus in the common wheat varieties with known information on their** ***Glu-D1*** **allele.**
(XLS)

Table S2 **Haplotype variation of** ***Glu-D1*** **locus in the common wheat lines from different countries.**
(XLS)

Table S3 ***Glu-D1*** **haplotypes detected in 355** ***Ae. tauschii*** **accessions.**
(XLS)

Table S4 ***Glu-D1*** **haplotypes detected in 215** ***T. spelta*** **accessions.**
(XLS)

Table S5 **The oligonucleotide primers used in this study.**
(DOC)

Table S6 **Estimation of approximate insertion time of** ***Sabrina-2*** **into** ***Glu-D1*** **locus.**
(DOC)

Table S7 **Number of nucleotide substitutions between the LTR sequences in the** ***Sabrina-2*** **elements resided in several** ***Glu-D1*** **haplotypes.**
(DOC)

Table S8 **Amino acid substitutions (S1 to S10) and indels (ID1 to ID5) between 1Dx5 and 1Dx2 of common wheat and their variations in the 20 1Dx subunits from** ***T. spelta*** **or** ***Ae. Tauschii.***
(DOC)

Table S9 **Divergence time estimates between two representative hexaploid wheat** ***1Dx*** **genes (** ***1Dx5*** **and** ***1Dx2*** **) and** ***Ae. tauschii 1Dx*** **genes.**
(DOC)

Methods S1 **Additional methods.**
(DOC)
