# Supplementary material for: Haplotype Variation of Glu-D1 Locus and the Origin of Glu-D1d Allele Conferring Superior End-Use Qualities in Common Wheat
Source: PLoS One. 2013 Sep 30;8(9):e74859. doi: 10.1371/journal.pone.0074859 (PMC3786984; doi:10.1371/journal.pone.0074859)
Supplement: Figure S1 — Validation of the chromosomal specificity of the seven newly developed Glu-D1 markers in this study. Genomic DNA samples were extracted from Chinese Spring (CS), the nulli-tetrasomic line N1DT1A (lacking 1D chromosome and associated Glu-D1 locus), the ditelosomic line of chromosomal arm 1DS (Dt1DS, lacking 1DL and associated Glu-D1 locus), three additional common wheat genotypes (Attila, Bobwhite and Kenong 199), two tetraploid wheat genotypes (Langdon and Cofa), and two Ae. tauschii accessions (AUS18913 and AL8/78). They were then used in genomic PCR with the primer pairs for each of the seven markers (Xms1, Xid1, Xrj1, Xrj2, Xms2, Xrj3, and Xrj4). These markers did not amplify products in tetraploid wheat genotypes, N1DT1A, and Dt1DS, confirming that they are D genome specific, and that their target sequences are located on chromosomal 1DL on which Glu-D1 resides. As anticipated, the seven markers showed polymorphisms in common wheat and between common wheat and Ae. tauschii. Lane M contains DNA size standard. The size (bp) of the amplified PCR fragments is shown on the left side of the graph. Items in the brackets indicate genome constitution of the examined wheat or Ae. tauschii genotypes. (PDF) [file pone.0074859.s001.pdf]

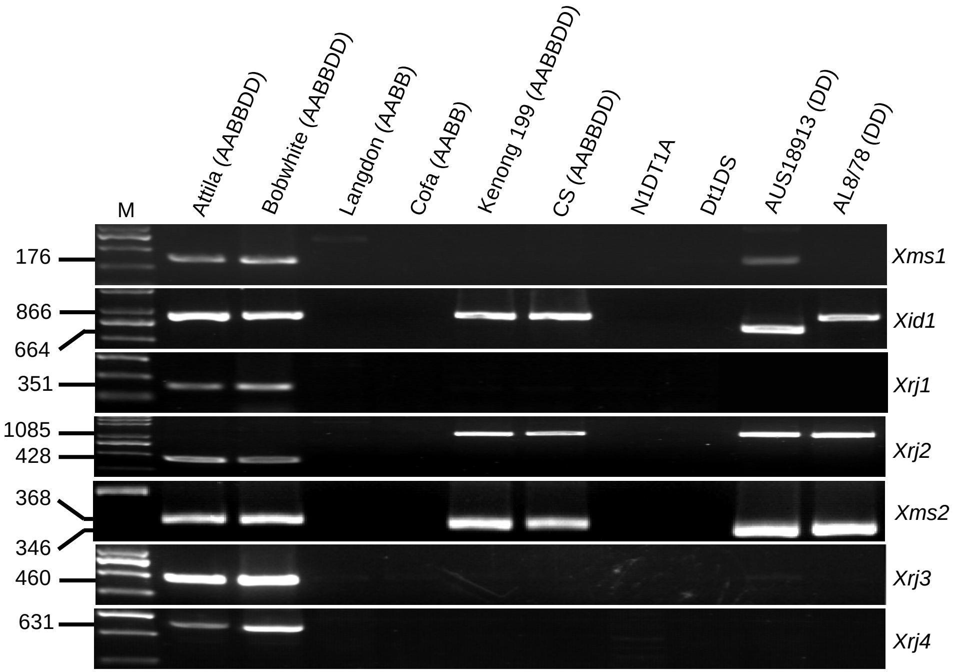
M
Attila (AABBDD)
Bobwhite (AABBDD)
Langdon (AABB)
Cofa (AABB)
Kenong 199 (AABBDD)
CS (AABBDD)
N1DT1A
Dt1DS
AUS18913 (DD)
AL8/78 (DD)
176
866
664
351
1085
428
368
346
460
631
Xms1
Xid1
Xrj1
Xrj2
Xms2
Xrj3
Xrj4

Figure S1 Validation of the chromosomal specificity of the seven newly developed *Glu-D1* markers in this study. Genomic DNA samples were extracted from Chinese Spring (CS), the nulli-tetrasomic line N1DT1A (lacking 1D chromosome and associated *Glu-D1* locus), the ditelosomic line of chromosomal arm 1DS (Dt1DS, lacking 1DL and associated *Glu-D1* locus), three additional common wheat genotypes (Attila, Bobwhite and Kenong 199), two tetraploid wheat genotypes (Langdon and Cofa), and two *Ae. tauschii* accessions (AUS18913 and AL8/78). They were then used in genomic PCR with the primer pairs for each of the seven markers (*Xms1*, *Xid1*, *Xrj1*, *Xrj2*, *Xms2*, *Xrj3*, and *Xrj4*). These markers did not amplify products in tetraploid wheat genotypes, N1DT1A, and Dt1DS, confirming that they are D genome specific, and that their target sequences are located on chromosomal 1DL on which *Glu-D1* resides. As anticipated, the seven markers showed polymorphisms in common wheat and between common wheat and *Ae. tauschii*. Lane M contains DNA size standard. The size (bp) of the amplified PCR fragments is shown on the left side of the graph. Items in the brackets indicate genome constitution of the examined wheat or *Ae. tauschii* genotypes.
