# Supplementary material for: Haplotype Variation of Glu-D1 Locus and the Origin of Glu-D1d Allele Conferring Superior End-Use Qualities in Common Wheat
Source: PLoS One. 2013 Sep 30;8(9):e74859. doi: 10.1371/journal.pone.0074859 (PMC3786984; doi:10.1371/journal.pone.0074859)
Supplement: Figure S3 — Detection of 1Dx and 1Dy subunits expressed by T. spelta and Ae. tauschii Glu-D1 Haplotypes. The 1Dx and 1Dy HMW-GSs in the two common wheat varieties, Bobwhite (expressing 1Dx5 and 1Dy10) and Chinese Spring (CS, expressing 1Dx2 and 1Dy12), and the T. spelta and Ae. tauschii accessions with different Glu-D1 haplotypes were revealed by SDS-PAGE analysis of seed protein extracts. 1Dx and 1Dy subunits specified by different T. spelta and Ae. tauschii Glu-D1 haplotypes were indicated by arrowheads and arrows, respectively. The protein band labeled by asterisk in H7 is a ω-gliadin protein. (PDF) [file pone.0074859.s003.pdf]

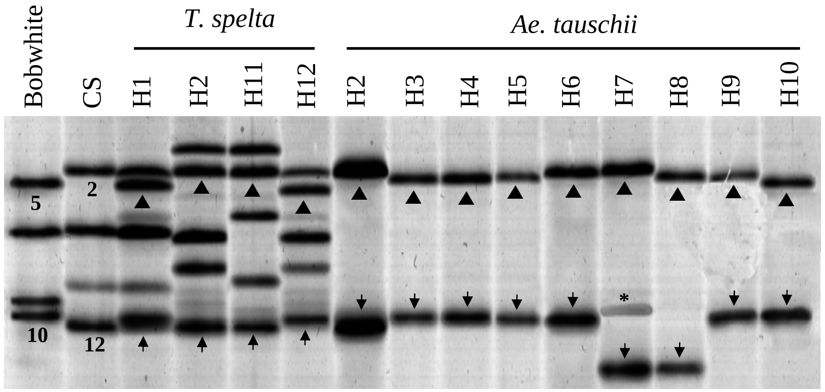

Figure S3 Detection of 1Dx and 1Dy subunits expressed by *T. spelta* and *Ae. tauschii Glu-D1* Haplotypes. The 1Dx and 1Dy HMW-GSs in the two common wheat varieties, Bobwhite (expressing 1Dx5 and 1Dy10) and Chinese Spring (CS, expressing 1Dx2 and 1Dy12), and the *T. spelta* and *Ae. tauschii* accessions with different *Glu-D1* haplotypes were revealed by SDS-PAGE analysis of seed protein extracts. 1Dx and 1Dy subunits specified by different *T. spelta* and *Ae. tauschii Glu-D1* haplotypes were indicated by arrowheads and arrows, respectively. The protein band labeled by asterisk in H7 is a ω-gliadin protein.
