# Supplementary material for: Haplotype Variation of Glu-D1 Locus and the Origin of Glu-D1d Allele Conferring Superior End-Use Qualities in Common Wheat
Source: PLoS One. 2013 Sep 30;8(9):e74859. doi: 10.1371/journal.pone.0074859 (PMC3786984; doi:10.1371/journal.pone.0074859)
Supplement: Figure S4 — Multiple alignment of the deduced amino acid sequences of 1Dx5 and 1Dx2 subunits from common wheat, and the 20 1Dx subunits from Ae. tauschii and T. spelta . The signal peptide is underlined, while the N- and C-terminal domains are labeled bold and bold italic, respectively. The repetitive domain is situated between the N- and C-terminal domains. The amino acid substitutions (S1 to S10) and indels (ID1 to ID5) between 1Dx5 and 1Dx2, and their variations in the 20 1Dx subunits from T. spelta and Ae. tauschii, are indicated. The four cysteine residues conserved among the 22 compared 1Dx subunits are marked by arrowheads. In addition, the cysteine reside unique to 1Dsx-TRI9883H1, 1Dsx-TRI16607H1 or 1Dsx-TRI5008H11 is boxed. (PDF) [file pone.0074859.s004.pdf]

S1

1Dx5	MAKRLVLFVAVVVALVALTVAEGEASEQLQCERELQELQERELKACQQVMDQQLRDISPECHPVVVSPVAGQYEQQIVVPPKGGSFYPGETTPPQQLQQRIFWGIPALLKRYYPSVTCPQQVSYYPGQAS
$1D^{s}x$-PI15865^{H1}	MAKRLVLFVAVVVALVALTVAEGEASEQLQCERELQELQERELKACQQVMDQQLRDISPECHPVVVSPVAGQYEQQIVVPPKGGSFYPGETTPPQQLQQRIFWGIPALLRRYYPSVTCPQQVSYYPGQAS
$1D^{s}x$-PI361813^{H1}	MAKRLVLFVAVVVALVALTVAEGEASEQLQCERELQELQERELKACQQVMDQQLRDISPECHPVVVSPVAGQYEQQIVVPPKGGSFYPGETTPPQQLQQRIFWGIPALLKRYYPSVTCPQQVSYYPGQAS
$1D^{s}x$-TRI9883^{H1}	MAKRLVLFVAVVVALVALTVAEGEASEQLQCERELQELQERELKACQQVMDQQLRDISPECHPVVVSPVAGQYEQQIVVPPKGGSFYPGETTPPQQLQQRIFWGIPALLKRYYPSVTCPQQVSYYPGQAS
$1D^{s}x$-TRI16607^{H1}	MAKRLVLFVAVVVALVALTVAEGEASEQLQCERELQELQERELKACQQVMDQQLRDISPECHPVVVSPVAGQYEQQIVAPPKGGSFYPGETTPPQQLQQRIFWGIPALLKRYYPSVTCPQQVSYYPGQAS
$1D^{s}x$-TRI16981^{H1}	MAKRLVLFVAVVVALVALTVAEGEASEQLQCERELQELQERELKACQQVMDQQLRDISPECHPVVVSPVAGQYEQQIVVPPKGGSFYPGETTPPQQLQQRIFWGIPALLKRYYPSVTCPQQVSYYPGQAS
$1D^{s}x$-PI347904^{H1}	MAKRLVLFVAVVVALVALTVAEGEASEQLQCERELQELQERELKACQQVMDQQLRDISPECHPVVVSPVAGQYEQQIVVPPKGGSFYPGETTPPQQLQQRIFWGIPALLKRYYPSVTCPQQVSYYPGQAS
$1D^{s}x$-PI348150^{H1}	MAKRLVLFVAVVVALVALTVAEGEASEQLQCERELQELQERELKACQQVMDQQLRDISPECHPVVVSPVAGQYEQQIVVPPKGGSFYPGETTPPQQLQQRIFWGIPALLKRYYPSVTCPQQVSYYPGQAS
$1D^{s}x$-PI348171^{H1}	MAKRLVLFVAVVVALVALTVAEGEASEQLQCERELQELQERELKACQRVMDQQLRDISPECHPVVVSPVAGQYEQQIVVPPKGGSFYPGETTPPQQLQQRIFWGIPALLRRYYPSVTCPQQVSYYPGQAS
$1D^{s}x$-TRI19057^{H12}	MAKRLVLFVAVVVALVALTVAEGEASEQLQCERELQELQERELKACQQVMDQQLRDISPECHPVVVSPVAGQYEQQIGVPPKGGSFYPGETTPPQQLQQRIFWGIPALLKRYYPSVTSPQQVSYYPGQAS
$1D^{t}x$-PI349047^{H9}	MAKRLVLFVAVVVALVALTVAEGEASEQQQCERELQELQERELKACQQVMDQQLRDISPECHPVVVSPVAGQYEQQIVVPPKGGSFYPGETTPPQQLQQRIFWGIPALLKRYYPSVTSPQQVSYYPGQAS
$1D^{t}x$-PI603223^{H10}	MAKRLVLFVAVVVALVALTVAEGEASEQLQCERELQELQERELKACQQVMDQQLRDISPECHPVVVSPVAGQYEQQIVVPPKGGSFYPGETTPPQQLQQRIFWGIPALLKRYYPSVTSPQQVSYYPGQAS
$1D^{t}x$-IG46663^{H3}	MAKRLVLFVAVVVALVALTVAEGEASEQLQCERELQELQERELKACQQVMDQQLRDISPECHPVVVSPVAGQYEQQIVVPPKGGSLYPGETTPPQQLQQRIFWGIPALLKRYYPSVTSPQQVSYYPGQAS
$1D^{t}x$-PI603224^{H4}	MAKRLVLFVAVVVALVALTVAEGEASEQLQCEHELQELQERELKACQQVMDQQLRDISPECHPVVVSPVAGQYEQQIVVPPKGGSFYPGETTPPQQLQQRIFWGIPTLLKRYYPSVTSPQQVSYYPGQAS
$1D^{t}x$-IG48561^{H5}	MAKRLVLFVAVVVALVALTVAEGEASEQLQCERELQELQERELKACQQVMDQQLRDISPECHPVVVSPVAGQYEQQIVVPPKGGSLYPGETTPPQQLQQRIFWGIPALLKRYYPSVTSPQQVSYYPGQAS
$1D^{t}x$-PI603236^{H6}	MAKRLVLFVAVVVALVALTVAEGEASEQLQCERELQELQERELKACQQVMDQQLRDISPECHPVVVSPVAGQYEQQIVVPPKGGSFYPGETTPPQQLQQRIFWGIPALLKRYYPSVTSPQQVSYYPGQAS
$1D^{t}x$-CIAE24^{H7}	MAKRLVLFVAVVVALVALTVAEGEASEQLQCERELQELQERELKACQQVMDQQLRDISPECHPVVVSPVAGQYEQQIVVPPKGGSFYPGETTPPQQLQQRIFWGIPALLKRYYPSVTSPQQVSYYPGQAS
$1D^{t}x$-TA2527^{H8}	MAKRLVLFVAVVVALVALTVAEGEASEQLQCERELQELQERELKACQQVMDQQLRDISPECHPVVVSPVAGQYEQQIVVPPKGGSFYPGETTPPQQLQQRIFWGIPALLKRYYPSVTSPQQVSYYPGQAS
$1D^{t}x$-PI511368^{H2}	MAKRLVLFVAVVVALVALTVAEGEASEQLQCERELQELQERELKACQQVMDQQLRDISPECHPVVVSPVAGQYEQQIVVPPKGGSFYPGEATPPQQLQQRIFWGIPALLKRYYPSVTSPQQVSYYPGQAS
$1D^{s}x$-PI348360^{H2}	MAKRLVLFVAVVVALVALTVAEGEASEQLQCERELQELQERELKACQQVMDQQLRDISPECHPVVVSPVAGQYEQQIVVPPKGGSFYPGETTPPQQLQQRIFWGIPALLKRYYPSVTSPQQVSYYPGQAS
$1D^{s}x$-TRI5008^{H11}	MAKRLVLFVAVVVALVALTVAEGEASEQLQCERELQELQERELKACQQVMDQQLRDISPECHPVVVSPVAGQYEQQIVVPPKGGSFYPGETTPPQQLQQRIFWGIPALLKRYYPSVTSPQQVSYYPGQAS
1Dx2	MAKRLVLFVAVVVALVALTVAEGEASEQLQCERELQELQERELKACQQVMDQQLRDISPECHPVVVSPVAGQYEQQIVVPPKGGSFYPGETTPPQQLQQRIFWGIPALLKRYYPSVTSPQQVSYYPGQAS
	********************************:**:********************:**:*******.************

ID1 S2 S3 S4 S5

1Dx5	PQRPGQGQQPGQGQQ------GYYPTSPQQPGQWQQPEQGQPRYYPTSPQQSGQLQQPAQGQQPGQGQQGQQPGQGQPGYYPTSSQLQPGQLQQPAQGQQGQQPGQAQQGQQPGQGQQPGQGQQGQQPGQ
$1D^{s}x$-PI15865^{H1}	PQRPGQGQQPGQGQQ------GYYPTSPQQPGQWQQPEQGQPRYYPTSPQQSGQLQQPAQGQQPGQGQQGQQPGQGQPGYYPTSSQLQPGQLQQPAQGQQGQQPGQGQQGQQPGQGQQPGQGQQGQQPGQ
$1D^{s}x$-PI361813^{H1}	PQRPGQGQQPGQGQQ------GYYPTSPQQPGRWQQPEQGQPRYYPTSPQQSGQLQQPAQGQQPGQGQQGQQPGQGQPGYYPTSSQLQPGQLQQPAQGQQGQQPGQGQQGQQPGQGQQPGQGQQGQQPGQ
$1D^{s}x$-TRI9883^{H1}	PQRPGQGQQPGQGQQ------GYYPTSPQQPGQWQQPEQGQPRYYPTSPQQSGQLQQPAQGQQPGQGQQGQQPGQGQPGYYPTSSQLQPGQLQQPAQGQQGQQPGQGQQGQQPGQGQQPGQGQQGQQPGQ
$1D^{s}x$-TRI16607^{H1}	PQRPGRGQQPGQGQQ------GYYPTSPQQPGQWQQPEQGQPRYYPTSPQQSGQLQQPAQGQQPGQGQQGQQPGQGQPGYHPTSSQLQLGQLQQPAQGQQGQQPGQGQQGQQPGQGQQPGQGQQGQQPGQ
$1D^{s}x$-TRI16981^{H1}	PQRPGQGQQPGQGQQ------GYYPTSPQQPGQWQQPEQGQPRYYPTSPQQSGQLQQPAQGQQPGQGQQGQQPGQGQPGYYPTSSQLQPGQLQQPAQGQQGQQPGQGQQGQQPGQGQRPGQGQQGQQPGQ
$1D^{s}x$-PI347904^{H1}	PQRPGQGQQPGQGQQ------GYYPTSPQQPGQWQQPEQGQPRYYPTSPQQSGQLQQPAQGQQPGQGQQGQQPGQGQPGYYPTSSQLQPGQLQQPAQGQQGQQPGQGQQGQQPGQGQQPGQGQQGQQPGQ
$1D^{s}x$-PI348150^{H1}	PQRPGQGQQPGQGQQ------GYYPTSPQQPGQWQQPEQGQPRYYPTSPQQSGQLRQPAQGQQPGQGQQGQQPGQGQPGYYPTSSQLQPGQLQQPAQGQQGQQPGQGQQGQQPGQGQQPGQGQQGQQPGQ
$1D^{s}x$-PI348171^{H1}	PQRPGQGQQPGQGQQ------GYYPTSPQQPGQWQQPEQGQPRYYPTSPQQSGQLQQPAQGQQPGQGQQGQQPGQGQPGYYPTSSQLQPGQLQQPAQGQQGQQPGQGQQGQQPGQGQQPGQGQQGQQPGQ
$1D^{s}x$-TRI19057^{H12}	PQRPGQGQQPGQGQQ------GYYPTSPQQPGQWQQPEQGQPRYYPTSPQQSGQLQQPAQGQQPGRGQQGQQPGQGQPGYYPTSSQLQPGQLQQPAQGQQGQQPGQGQQGQQPGQGQQPGQGQQGQQPGQ
$1D^{t}x$-PI349047^{H9}	PQRPGQGQQPGQGQQSGQGQQGYYPTSPQQPGQWQQPEQGQPGYYPTSPQQSGQLQQPAQGQQPGQGQQGQQPGQGQPGYYPTSSQLQPGQLQQPAQGQQGQQPGQGQQGQQPGQGQQPGQGQQGQQPGQ
$1D^{t}x$-PI603223^{H10}	PQRPGQGQQPGQGQQSGQGQQGYYPTSPQQPGQWQQPEQGQPGYYPTSPQQSGQLQRPAQGQQPGQGQQGQQPGQGQPGYYPTSSQLQPGQLQQPAQGQQGQQPGQGQQGQQPGQGQQPGQGQQGQQPGQ
$1D^{t}x$-IG46663^{H3}	PQRPGQGQQPGQGQQSGQGQQGYYPTSPQQPGQWQQPEQGQPGYYPTSPQQPGQLQQPAQGQQPGQGQQGQQPGQGQPGYYPTSSQLQPGQLQQPAQGQQGQQPGQGQQGQQPGQGQQPGQGQQGQQPGQ
$1D^{t}x$-PI603224^{H4}	PQRPGQGQQPGQGQQSGQGQQGYYPTSPQQPGQWQQPEQGQPGYYPTSPQQPGQLQQPAQGQQPGQGQQGQQPGQGQPGYYPTSSQLQPGQLQQPAQGQQGQQPGQGQQGQQPGQGQQPGQGQQGQQPGQ
$1D^{t}x$-IG48561^{H5}	PQRPGQGQQPGQGQQSGQGQQGYYPTSPQQPGQWQQPEQGQPGYYPTSPQQPGQLQQPAQGQQPGQGQQGQQPGQGQPGYYPTSSQLQPGQLQQPAQGQQGQQPGQGQQGQQPGQGQQPGQGQQGQQPGQ
$1D^{t}x$-PI603236^{H6}	PQRPGQGQQPGQGQQSGQGQQGYYPTSPQQPGQWQQPEQGQPGYYPTSPQQPGQLQQPAQGQQPGQGQQGRQPGQGQPGYYPTSSQLQPGQLQQPAQGQQGQQPGQGQQGQQPGQGQQPGQGQQGQQPGQ
$1D^{t}x$-CIAE24^{H7}	PQRPGQGQQPGQGQQSGQGQQGYYPTSPQQPGQWQQPEQGQPGYYPTSPQQPGQLQQPAQGQQPGQGQQGRQPGQGQPGYYPTSSQLQPGQLQQPAQGQQGQQPGQGQQGQQPGQGQQSGQGQQGQQPGQ
$1D^{t}x$-TA2527^{H8}	PQRPGQGQQPGQGQQSGQGQQGYYPTSPQQPGQWQQPEQGQPGYYPTSPQQPGQLQQPAQGQQPGQGQQGRQPGQGQPGYYPTSSQLQPGQLQQPAQGQQGQQPGQGQQGQQPGQGQQSGQGQQGQQPGQ
$1D^{t}x$-PI511368^{H2}	PQRPGQGQQPGQGQQSGQGQQGYYPTSPQQPGQWQQPEQGQPGYYPTSPQQPGQLQQPAQGQQPGQGQQGRQPGQGQPGYYPTSSQLQPGQLQQPAQGQQGQQPGQGQQGQQPGQGQQPGQGQQGQQPGQ
$1D^{s}x$-PI348360^{H2}	PQRPGQGQQPGQGQQSGQGQQGYYPTSPQQPGQWQQPEQGQPGYYPTSPQQPGQLQQPAQGQQPGQGQQGRQPGQGQPGYYPTSSQLQPGQLQQPAQGQQGQQPGQGQQGQQPGQGQQPGQGQQGQQPGQ
$1D^{s}x$-TRI5008^{H11}	PQRPGQGQQPGQGQQSGQGQQGYYPTSPQQPGQWQQPEQGQPGYYPTSPQQPGQLQQPAQGQQPGQGQQGRQPGQGQPGYYPTSSQLQPGQLQQPAQGQQGQQPGQGQQGQQPGQGQQPGQGQQGQQPGQ
1Dx2	PQRPGQGQQPGQGQQSGQGQQGYYPTSPQQPGQWQQPEQGQPGYYPTSPQQPGQLQQPAQGQQPGQGQQGRQPGQGQPGYYPTSSQLQPGQLQQPAQGQQGQQPGQGQQGQQPGQGQQPGQGQQGQQPGQ
	*****:********* ***********:********* ********.***::********:****:*********:******* *****************.**********:.***********

```
                                                                                                              S6
1Dx5              GQQPGQGQQGQQLGQGQQGYYPTSLQQSGQGQPGYYPTSLQQLGQGQSGYYPTSPQQPGQGQQPGQLQQPAQGQQPGQGQQGQQPGQGQQGQQPGQGQQPGQGQPGYYPTSPQQSGQGQPGYYPTSSQQP
1Dsx-PI15865H1    GQQPGQGQQGQQLGQGQQGYYPTSLQQSGQGQPGYYPTSLQQLGQGQSGYYPTSPQQPGQGQQPGQLQQPAQGQQPGQGQQGQQPGQGQQGQQPGQGQQPGQGQPGYYPTSPQQSGQGQPGYYPTSSQQP
1Dsx-PI361813H1   GQQPGQGQQGQQLGQGQQGYYPTSLQQSGQGQPGYYPTSLQQLGQGQSGYYPTSPQQPGQGQQPGQLQQPAQGQQPGQGQQGQQPGQGQQGQQPGQGQQPGQGQPGYYPTSPQQSGQGQPGYYPTSSQQP
1Dsx-TRI9883H1    GQQPGQGQQGQQLGQGQQGYYPTSLQQSGQGQPGYYPTSLQQLGQGQSGYYPTSPQQPGQGQQPGQLQQPAQGQQPGQGQQGQQPGQGQQGQQPGQGQQPGQGQPGYYPTSPQQSGQGQPGYYPTSSQQP
1Dsx-TRI16607H1   GQQPGQGQQGQQLRQGQQGYYPTSLQQSGQGQPGYYPTSLQQLGQGQSGYYPTSPQQPGQGQQPGQLQQPAQGQQPGQGQQGQQPGQGQQGQQPGQGQQPGQGQPGCYPTSPQQSGQGQPGYYPTSSQQP
1Dsx-TRI16981H1   GQQPGQGQQGQQLGQGQQGYYPTSLQQSGQGQPGYYPTSLQQLGQGQSGYYPTSPQQPGQGQQPGQLQQPAQGQQPGQGQQGQQPGQGQQGQQPGQGRQPGQGQPGYYPTSPQQSGQEQPGYYPTSSQQP
1Dsx-PI347904H1   GQQPGQGQQGQQLGQGQQGYYPTSLQQSGQGQPGYYPTSLQQLGQGQSGYYPTSPQQPGQGQQPGQLQQPAQGQQPGQGQQGQQPGQGQQGQQPGQGQQPGQGQPGYYPTSPQQSGQGQPGYYPTSSQQP
1Dsx-PI348150H1   GQQPGQGQQGQQLGQGQQGYYPTSLQQSGQGQPGYYPTSLQQLGQGQSGYYPTSPQQPGQGQQPGQLQQPAQGQQPGQGQQGQQPGQGQQGQQPGQGQQPGQGQPGYYPTSPQQSGQGQPGYYPTSSQQP
1Dsx-PI348171H1   GQQPGQGQQGQQLGQGQQGYYPTSLQQSGQGQPGYYPTSLQQLGQGQSGYYPTSPQQPGQGQQPGQLQQPAQGQQPGQGQQGQQPGQGQQGQQPGQGQQPGQGQPGYYPTSPQQSGQGQPGYYPTSSQQP
1Dsx-TRI19057H12  GQQPGQGQQGQQLGQGQQGYYPTSLQQSGQGQPGYYPTSLQQLGQGQSGYYPTSPQQPGQGQQPGQLQQPAQGQQPGQGQQGQQPGQGQQGQQPGQGQQPGQGQPGYYPTSPQQSGQGQPGYYPTSSQQP
1Dtx-PI349047H9   GQQPGQGQQGQQLGQGQQGYYPTSLQQSGQGQPGYYPTSLQQLGQGQSGYYPTSPQQPGQGQQPGPLQQPAQGQQPGQGQQGRQPGQGQQGQQPGQGQQPGQGQPGYYPTSPQQSGQGQPGYYPTSSQQP
1Dtx-PI603223H10  GQQPGQGQQGQQLGQGQQGYYPTSLQQSGQGQPGYYPTSLQQLGQGQSGYYPTSPQQPGQGQQPGQLQQPAQGQQPGQGQQGRQPGQGQQGQQPGQGQQPGQGQPGYYPTSPQQSGQGQPGYYPTSSQQP
1Dtx-IG46663H3    GQQPGQGQQGQQLGQGQQGYYPTSLQQSGQGQPGYYPTSLQQLGQGQSGYYPTSPQQPGQGQQLGQLQQPAQGQQPGQGQQGQQPGQGQQGQQPGQGQQPGQGQPGYYPTSPQQSGQGQPGYYPTSSQQP
1Dtx-PI603224H4   GQQPGQGQQGQQLGQGQQGYYPTSLQQSGQGQPGYYPTSLQQLGQGQSGYYPTSPQQPGQGQQLGQLQQPAQGQQPGQGQQGQQPGQGQQGQQPGQGQQPGQGQPGYYPTSPQQSGQGQPGYYPTSSQQP
1Dtx-IG48561H5    GQQPGQGQQGQQLGQGQQGYYPTSLQQSGQGQPGYYPTSLQQLGQGQSGYYPTSPQQPGQGQQLGQLQQPAQGQQPGQGQQGQQPGQGQQGQQPGQGQQPGQGQPGYYPTSPQQSGQGQPGYYPTSSQQP
1Dtx-PI603236H6   GQQPGQGQQGQQLGQGQQGYYPTSLQQSGQGQPGYYPTSLQQLGQGQSGYYPTSPQQPGQGQQPGQLQQPAQGQQPGQGQQGQQPGQGQQGQQPGQGQQPGQGQPGYYPTSPQQSGQGQPGYYPTSSQQP
1Dtx-CIAE24H7     GQQPGQGQQGQQLGQGQQGYYPTSLQQSGQGQPGYYPTSLQQLGQGQSGYYPTSPQQPGQGQQPGQLQQPAQGQQPGQGQQGQQPGQGQQGQQPGQGQQPGQGQPGYYPTSPQQSGQGQPGYYPTSSQQP
1Dtx-TA2527H8     GQQPGQGQQGQQLGQGQQGYYPTSLQQSGQGQPGYYPTSLQQLGQGQSGYYPTSPQQPGQGQQPGQLQQLAQGQQPGQGQQGQQPGQGQQGQQPGQGQQPGQGQPGYYPTSPQQSGQGQPGYYPTSSQQP
1Dtx-PI511368H2   GQQPGQGQQGQQLGQGQQGYYPTSLQQSGQGQPGYYPTSLQQLGQGQSGYYPTSPQQPGQGQQPGQLQQPAQGQQPEQGQQGQQPGQGQQGQQPGQGQQPGQGQPGYYPTSPQQSGQGQPGYYPTSSQQP
1Dsx-PI348360H2   GQQPGQGQQGQQLGQGQQGYYPTSLQQSGQGQPGYYPTSLQQLGQGQSGYYPTSPQQPGQGQQPGQLQQPAQGQQPEQGQQGQQPGQGQQGQQPGQGQQPGQGQPGYYPTSPQQSGQGQPGYYPTSSQQP
1Dsx-TRI5008H11   GQQPGQGQQGQQLGQGQQGYYPTSLQQSGQGQPGYYPTSLQQLGQGQSGYCPTSPQQPGQGQQPGQLQQPAQGQQPEQGQQGQQPGQGQQGQQPGQGQQPGQGQPGYYPTSPQQSGQGQPGYYPTSSQQP
1Dx2              GQQPGQGQQGQQLGQGQQGYYPTSLQQSGQGQPGYYPTSLQQLGQGQSGYYPTSPQQPGQGQQPGQLQQPAQGQQPEQGQQGQQPGQGQQGQQPGQGQQPGQGQPGYYPTSPQQSGQGQPGYYPTSSQQP
                  ************* ************************************ ************ * *** ****** *****:**************:******** ********** ************

                                                           S7
1Dx5              TQSQQPGQGQQGQQVGQGQQAQQPGQGQQPGQGQPGYYPTSPQQSGQGQPGYYLTSPQQSGQGQQPGQLQQSAQGQKGQQPGQGQQPGQGQQGQQPGQGQQGQQPGQGQPGYYPTSPQQSGQGQQPGQWQ
1Dsx-PI15865H1    TQSQQPGQGQQGQQVGQGQQAQQPGQGQQPGQGQPGYYPTSPQQSGQGQPGYYLTSPQQSGQGQQPGQLQQSAQGQKGQQPGQGQQPGQGQQGQQPGQGQQGQQPGQGQPGYYPTSPQQSGQGQQPGQWQ
1Dsx-PI361813H1   TQSQQPGQGQQGQQVGQGQQAQQPGQGQQPGQGQPGYYPTSPQQSGQGQPGYYLTSPQQSGQGQQPGQLQQSAQGQKGQQPGQGQQPGQGQQGQQPGQGQQGQQPGQGQPGYYPTSPQQSGQGQQPGQWQ
1Dsx-TRI9883H1    TQSQQPGQGQQGQQVGQGQQAQQPGQGQQPGQGQPGYYPTSPQQSGQGQPGYYLTSPQQSGQGQQPGQLQQSAQGQKGQQPGQGQQPGQGQQGQQPGQGQQGQQPGQGQPGYYPTSPQQSGQGQQPGQWQ
1Dsx-TRI16607H1   TQSQQPGQGQQGQQVGQGQQAQQPGQGQQPGQGQPGYYPTSPQQSGQGQPGYYLTSPQQSGQGQQPGQLQQSAQGQKGQQPGQGQQPGQGQQGQQPGQGQQGQQPGQGQPGYYPTSPQQSGQGQQPGQWQ
1Dsx-TRI16981H1   TQSQQPGQGQQGQQVGQGQQAQQPGQGQQPGQGQPGYYPTSPQQSGQGQPGYYLTPPQQSGQGQQPGQLQQSAQGQKGQQPGQGQQPGQGQQGQQPGQGQQGQQPGQGQPGYYPTSPQQSGQGQQPGQWQ
1Dsx-PI347904H1   TQSQQPGQGQQGQRVGQGQQAQQPGQGQQPGQGQPGYYPTSPQQSGQGQPGYYLTSPQQSGQGQQPGQLQQSAQGQKGQQPGQGQQPGQGQQGQQPGQGQQGQQPGQGQPGYYPTSPQQSGQGQQPGQWH
1Dsx-PI348150H1   TQPQQPGQGQQGQQVGQGQQAQQPGQGQQPGQGQPGYYPTSPQQSGQGQPGYYLTSPQQSGQGQQPGQLQQSAQGQKGQQPGQGQQPGQGQQGQQPGQGQQGQQPGQGQPGYYPTSPQQSGQGQQPGQWQ
1Dsx-PI348171H1   TQSQQPGQGQQGQQVGQGQQAQQPGQGQQPGQGQPGYYPTSPQQSGQGQPGYYLTSPQQSGQGQQPGQLQQSAQGQKGQQPGQGQQPGQGQQGQQPGQGQQGQQPGQGQPGYYPTSPQQSGQGQQPGQWE
1Dsx-TRI19057H12  TQSQQPGQGQQGQQVGQGQQAQQPGQGQQPGQGQPGYYPTSPQQSGQGQPGYYLTSSQQSGQGQQPGQLQQSAQGQKGQQPGQGQQPGQGQQGQQPGQGQQGQQPGQGQPGYYPISPQQSGQEQQPGQWQ
1Dtx-PI349047H9   TQSQQPGQGQRGQQVGQGQQAQQPGQGQQPGQGQPGYYPTSPQQSGQGQPGYYLTSPQQSGQGQQPGQLQQSAQGQKGQQPGQGQQPGQGQQGQQPGQGQQGQQPGQGQPGYYPTSPQQSGQGQQPGQWQ
1Dtx-PI603223H10  TQSQQPGQGQQGQQVGQGQQAQQPGQGQQPGQGQPGYYPTSPQQSGQGQPGYYLTSPQQSGQGQQPGQLQQSAQGQKGQQPGQGQQPGQGQQGQQPGQGQQGQQPGQGQPGYYPTSPQQSGQGQQPGQWQ
1Dtx-IG46663H3    TQSQQPGQGQQGQQVGQGQQAQQPGQGQQPGQGQPGYYPTSPQQSGQGQPGYYLTSPQQSGQGQQPGQLQQSAQGQKGQQPRQGQQPGQGQQGQQPGQGQQGQQPGQGQPGYYPTSPQQSGQGQQPGQWQ
1Dtx-PI603224H4   TQSQQPGQGQQGQQVGQGQQAQQPGQGQQPGQGQPGYYPTSPQQSGQGQPGYYLTSPQQSGQGQQPGQLQQSAQGQKGQQPGQGQQPGQGQQGQQPGQGQQGQQPGQGQPGYYPTSPQQSGQGQQPGQWQ
1Dtx-IG48561H5    TQSQQPGQGQQGQQVGQGQQAQQPGQGQQPGQGQPGYYPTSPQQSGQGQPGYYLTSPQQSGQGQQPGQLQQSAQGQKGQQPRQGQQPGQGQQGQQPGQGQQGQQPGQGQPGYYPTSPQQSGQGQQPGQWQ
1Dtx-PI603236H6   TQSQQPGQGQQGQQVGQGQQAQQPGQGQQPGQGQPGYYPTSPQQSGQGQPGYYLTSPQQSGQGQQPGQLQQSAQGQKGQQPGQGQQPGQGQQGQQPGQGQQGQQPGQGQPGYYPTSPQQSGQGQQPGQWQ
1Dtx-CIAE24H7     TQSQQPGQGQQGQQVGQGQQAQQPRQGQQPGQGQPGYYPTSPQQSGQGQPGYYLTSPQQSGQGQQPGQLQQSAQGQKGQQPGQGQQPGQGQQGQQPGQGQQGQQPGQGQPGYYPTSPQQSGQGQQPGQWQ
1Dtx-TA2527H8     TQSQQPGQGQQGQQVGQGQQAQQPRQGQQPGQGQPGYYPTSPQQSGQGQPGYYLTSPQQSGQGQQPGQLQQSAQGQKGQQPGQGQQPGQGQQGQQPGQGQQGQQPGQGQPGYYPTSPQQSGQGQQPGQWQ
1Dtx-PI511368H2   TQSQQPGQGQQGQQVGQGQQAQQPGQGQQPGQGQPGYYPTSPLQSGQGQPGYYLTSPQQSGQGQQPGQLQQSAQGQKGQQPGQGQQPGQGQQGQQPGQGQQGQQPGQGQPGYYPTSPQQSGQGQQPGQWQ
1Dsx-PI348360H2   TQSQQPGQGQQGQQVGQGQQAQQPGQGQQPGQGQPGYYPTSPLQSGQGQPGYYLTSPQQSGQGQQPGQLQQSAQGQKGQQPGQGQQPGQGQQGQQPGQGQQGQQPGQGQPGYYPTSPQQSGQGQQPGQWQ
1Dsx-TRI5008H11   TQSQQPGQGQQGQQVGQGQQAQQPGQGQQPGQGQPGYYPTSPLQSGQGQPGYYLTSPQQSGQGQQPGQLQQSAQGQKGQQPGQGQQPGQGQQGQQPGQGQQGQQPGQGQPGYYPTSPQQSGQGQQPGQWQ
1Dx2              TQSQQPGQGQQGQQVGQGQQAQQPGQGQQPGQGQPGYYPTSPLQSGQGQPGYYLTSPQQSGQGQQPGQLQQSAQGQKGQQPGQGQQPGQGQQGQQPGQGQQGQQPGQGQPGYYPTSPQQSGQGQQPGQWQ
                  **.*******:**:********** ***************** ************..************************ ***********************************************.
```

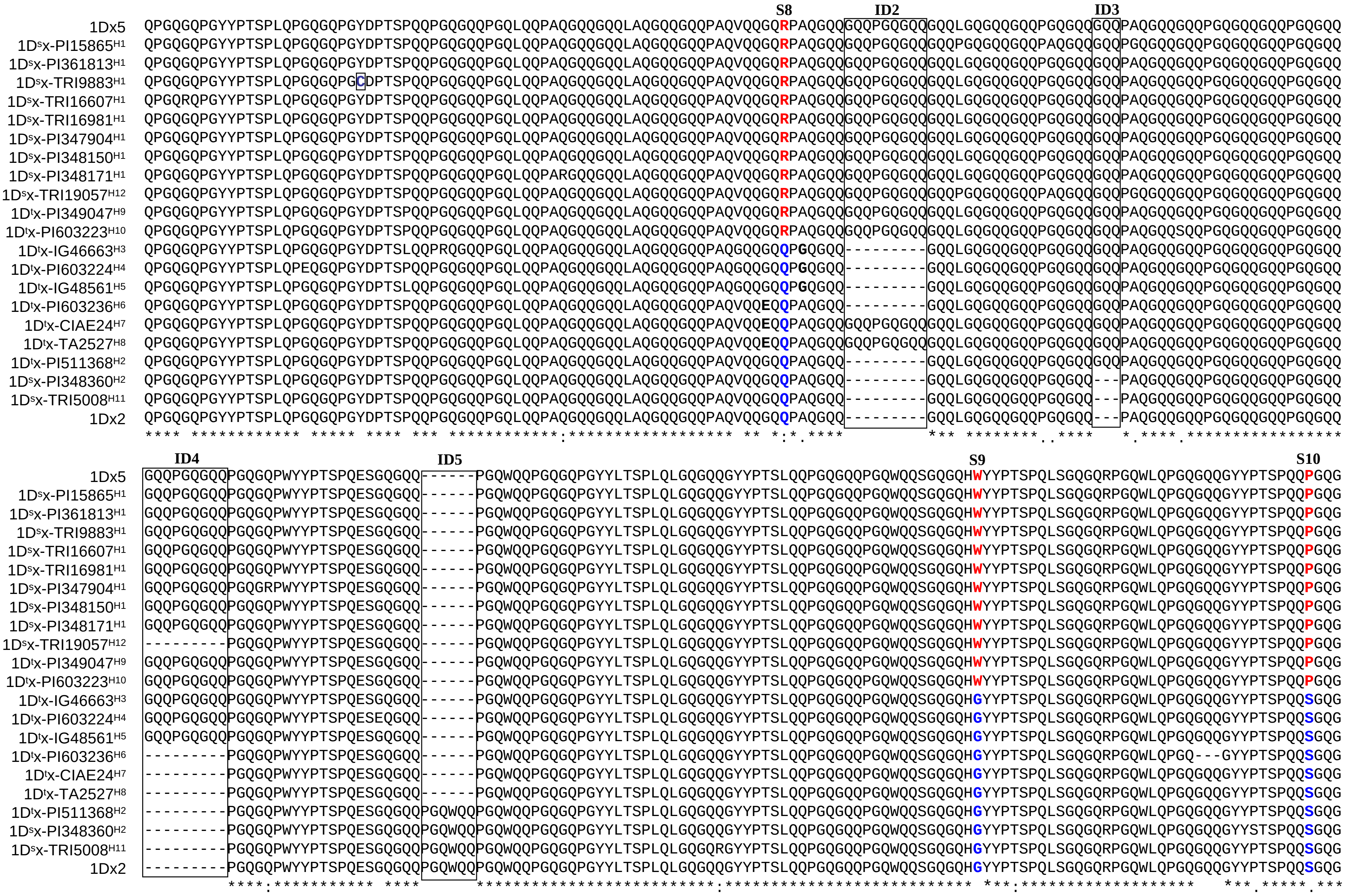
S8 ID2 ID3
1Dx5 QPGQGQPGYYPTSPLQPGQGQPGYDPTSPQQPGQGQQPGQLQQPAQGQQGQQLAQGQQGQQPAQVQQGQRPAQGQQGQQPGQGQQGQQLGQGQQGQQPGQGQQGQQPAQGQQGQQPGQGQQGQQPGQGQQ
1D^{s}x-PI15865^{H1} QPGQGQPGYYPTSPLQPGQGQPGYDPTSPQQPGQGQQPGQLQQPAQGQQGQQLAQGQQGQQPAQVQQGQRPAQGQQGQQPGQGQQGQQPGQGQQGQQPAQGQQGQQPGQGQQGQQPGQGQQGQQPGQGQQ
1D^{s}x-PI361813^{H1} QPGQGQPGYYPTSPLQPGQGQPGYDPTSPQQPGQGQQPGQLQQPAQGQQGQQLAQGQQGQQPAQVQQGQRPAQGQQGQQPGQGQQGQQLGQGQQGQQPGQGQQGQQPAQGQQGQQPGQGQQGQQPGQGQQ
1D^{s}x-TRI9883^{H1} QPGQGQPGYYPTSPLQPGQGQPGCDPTSPQQPGQGQQPGQLQQPAQGQQGQQLAQGQQGQQPAQVQQGQRPAQGQQGQQPGQGQQGQQLGQGQQGQQPGQGQQGQQPAQGQQGQQPGQGQQGQQPGQGQQ
1D^{s}x-TRI16607^{H1} QPGQRQPGYYPTSPLQPGQGQPGYDPTSPQQPGQGQQPGQLQQPAQGQQGQQLAQGQQGQQPAQVQQGQRPAQGQQGQQPGQGQQGQQLGQGQQGQQPGQGQQGQQPAQGQQGQQPGQGQQGQQPGQGQQ
1D^{s}x-TRI16981^{H1} QPGQGQPGYYPTSPLQPGQGQPGYDPTSPQQPGQGQQPGQLQQPAQGQQGQQLAQGQQGQQPAQVQQGQRPAQGQQGQQPGQGQQGQQLGQGQQGQQPGQGQQGQQPAQGQQGQQPGQGQQGQQPGQGQQ
1D^{s}x-PI347904^{H1} QPGQGQPGYYPTSPLQPGQGQPGYDPTSPQQPGQGQQPGQLQQPAQGQQGQQLAQGQQGQQPAQVQQGQRPAQGQQGQQPGQGQQGQQLGQGQQGQQPGQGQQGQQPAQGQQGQQPGQGQQGQQPGQGQQ
1D^{s}x-PI348150^{H1} QPGQGQPGYYPTSPLQPGQGQPGYDPTSPQQPGQGQQPGQLQQPAQGQQGQQLAQGQQGQQPAQVQQGQRPAQGQQGQQPGQGQQGQQLGQGQQGQQPGQGQQGQQPAQGQQGQQPGQGQQGQQPGQGQQ
1D^{s}x-PI348171^{H1} QPGQGQPGYYPTSPLQPGQGQPGYDPTSPQQPGQGQQPGQLQQPARGQQGQQLAQGQQGQQPAQVQQGQRPAQGQQGQQPGQGQQGQQLGQGQQGQQPGQGQQGQQPAQGQQGQQPGQGQQGQQPGQGQQ
1D^{s}x-TRI19057^{H12} QPGQGQPGYYPTSPLQPGQGQPGYDPTSPQQPGQGQQPGQLQQPAQGQQGQQLAQGQQGQQPAQVQQGQRPAQGQQGQQPGQGQQGQQPGQGQQGQQPAQGQQGQQPGQGQQGQQPGQGQQGQQPGQGQQ
1D^{t}x-PI349047^{H9} QPGQGQPGYYPTSPLQPGQGQPGYDPTSPQQPGQGQQPGQLQQPAQGQQGQQLAQGQQGQQPAQVQQGQRPAQGQQGQQPGQGQQGQQLGQGQQGQQPGQGQQGQQPAQGQQGQQPGQGQQGQQPGQGQQ
1D^{t}x-PI603223^{H10} QPGQGQPGYYPTSPLQPGQGQPGYDPTSPQQPGQGQQPGQLQQPAQGQQGQQLAQGQQGQQPAQVQQGQRPAQGQQGQQPGQGQQGQQLGQGQQGQQPGQGQQGQQPAQGQQSQQPGQGQQGQQPGQGQQ
1D^{t}x-IG46663^{H3} QPGQGQPGYYPTSPLQPGQGQPGYDPTSLQQPRQGQQPGQLQQPAQGQQGQQLAQGQQGQQPAQGQQGQQPGQGQQ---------GQQLGQGQQGQQPGQGQQGQQPAQGQQGQQPGQGQQGQQPGQGQQ
1D^{t}x-PI603224^{H4} QPGQGQPGYYPTSPLQPEQGQPGYDPTSPQQPGQGQQPGQLQQPAQGQQGQQLAQGQQGQQPAQGQQGQQPGQGQQ---------GQQLGQGQQGQQPGQGQQGQQPAQGQQGQQPGQGQQGQQPGQGQQ
1D^{t}x-IG48561^{H5} QPGQGQPGYYPTSPLQPGQGQPGYDPTSLQQPGQGQQPGQLQQPAQGQQGQQLAQGQQGQQPAQGQQGQQPGQGQQ---------GQQLGQGQQGQQPGQGQQGQQPAQGQQGQQPGQGQQGQQPGQGQQ
1D^{t}x-PI603236^{H6} QPGQGQPGYYPTSPLQPGQGQPGYDPTSPQQPGQGQQPGQLQQPAQGQQGQQLAQGQQGQQPAQVQQEQQPAQGQQ---------GQQLGQGQQGQQPGQGQQGQQPAQGQQGQQPGQGQQGQQPGQGQQ
1D^{t}x-CIAE24^{H7} QPGQGQPGYYPTSPLQPGQGQPGYDPTSPQQPGQGQQPGQLQQPAQGQQGQQLAQGQQGQQPAQVQQEQQPAQGQQGQQPGQGQQGQQLGQGQQGQQPGQGQQGQQPAQGQQGQQPGQGQQGQQPGQGQQ
1D^{t}x-TA2527^{H8} QPGQGQPGYYPTSPLQPGQGQPGYDPTSPQQPGQGQQPGQLQQPAQGQQGQQLAQGQQGQQPAQVQQEQQPAQGQQGQQPGQGQQGQQLGQGQQGQQPGQGQQGQQPAQGQQGQQPGQGQQGQQPGQGQQ
1D^{t}x-PI511368^{H2} QPGQGQPGYYPTSPLQPGQGQPGYDPTSPQQPGQGQQPGQLQQPAQGQQGQQLAQGQQGQQPAQVQQGQQPAQGQQ---------GQQLGQGQQGQQPGQGQQGQQPAQGQQGQQPGQGQQGQQPGQGQQ
1D^{s}x-PI348360^{H2} QPGQGQPGYYPTSPLQPGQGQPGYDPTSPQQPGQGQQPGQLQQPAQGQQGQQLAQGQQGQQPAQVQQGQQPAQGQQ---------GQQLGQGQQGQQPGQGQQ---PAQGQQGQQPGQGQQGQQPGQGQQ
1D^{s}x-TRI5008^{H11} QPGQGQPGYYPTSPLQPGQGQPGYDPTSPQQPGQGQQPGQLQQPAQGQQGQQLAQGQQGQQPAQVQQGQQPAQGQQ---------GQQLGQGQQGQQPGQGQQ---PAQGQQGQQPGQGQQGQQPGQGQQ
1Dx2 QPGQGQPGYYPTSPLQPGQGQPGYDPTSPQQPGQGQQPGQLQQPAQGQQGQQLAQGQQGQQPAQVQQGQQPAQGQQ---------GQQLGQGQQGQQPGQGQQ---PAQGQQGQQPGQGQQGQQPGQGQQ
**** ************ ***** **** *** ************:***************** ** *:*.****   *** ********..****   *.****.*****************
ID4 ID5 S9 S10
1Dx5 GQQPGQGQQPGQGQPWYYPTSPQESGQGQQ------PGQWQQPGQGQPGYYLTSPLQLGQGQQGYYPTSLQQPGQGQQPGQWQQSGQGQHWYYPTSPQLSGQGQRPGQWLQPGQGQQGYYPTSPQQPGQG
1D^{s}x-PI15865^{H1} GQQPGQGQQPGQGQPWYYPTSPQESGQGQQ------PGQWQQPGQGQPGYYLTSPLQLGQGQQGYYPTSLQQPGQGQQPGQWQQSGQGQHWYYPTSPQLSGQGQRPGQWLQPGQGQQGYYPTSPQQPGQG
1D^{s}x-PI361813^{H1} GQQPGQGQQPGQGQPWYYPTSPQESGQGQQ------PGQWQQPGQGQPGYYLTSPLQLGQGQQGYYPTSLQQPGQGQQPGQWQQSGQGQHWYYPTSPQLSGQGQRPGQWLQPGQGQQGYYPTSPQQPGQG
1D^{s}x-TRI9883^{H1} GQQPGQGQQPGQGQPWYYPTSPQESGQGQQ------PGQWQQPGQGQPGYYLTSPLQLGQGQQGYYPTSLQQPGQGQQPGQWQQSGQGQHWYYPTSPQLSGQGQRPGQWLQPGQGQQGYYPTSPQQPGQG
1D^{s}x-TRI16607^{H1} GQQPGQGQQPGQGQPWYYPTSPQESGQGQQ------PGQWQQPGQGQPGYYLTSPLQLGQGQQGYYPTSLQQPGQGQQPGQWQQSGQGQHWYYPTSPQLSGQGQRPGQWLQPGQGQQGYYPTSPQQPGQG
1D^{s}x-TRI16981^{H1} GQQPGQGQQPGQGQPWYYPTSPQESGQGQQ------PGQWQQPGQGQPGYYLTSPLQLGQGQQGYYPTSLQQPGQGQQPGQWQQSGQGQHWYYPTSPQLSGQGQRPGQWLQPGQGQQGYYPTSPQQPGQG
1D^{s}x-PI347904^{H1} GQQPGQGQQPGQGRPWYYPTSPQESGQGQQ------PGQWQQPGQGQPGYYLTSPLQLGQGQQGYYPTSLQQPGQGQQPGQWQQSGQGQHWYYPTSPQLSGQGQRPGQWLQPGQGQQGYYPTSPQQPGQG
1D^{s}x-PI348150^{H1} GQQPGQGQQPGQGQPWYYPTSPQESGQGQQ------PGQWQQPGQGQPGYYLTSPLQLGQGQQGYYPTSLQQPGQGQQPGQWQQSGQGQHWYYPTSPQLSGQGQRPGQWLQPGQGQQGYYPTSPQQPGQG
1D^{s}x-PI348171^{H1} GQQPGQGQQPGQGQPWYYPTSPQESGQGQQ------PGQWQQPGQGQPGYYLTSPLQLGQGQQGYYPTSLQQPGQGQQPGQWQQSGQGQHWYYPTSPQLSGQGQRPGQWLQPGQGQQGYYPTSPQQPGQG
1D^{s}x-TRI19057^{H12} ---------PGQGQPWYYPTSPQESGQGQQ------PGQWQQPGQGQPGYYLTSPLQLGQGQQGYYPTSLQQPGQGQQPGQWQQSGQGQHWYYPTSPQLSGQGQRPGQWLQPGQGQQGYYPTSPQQPGQG
1D^{t}x-PI349047^{H9} GQQPGQGQQPGQGQPWYYPTSPQESGQGQQ------PGQWQQPGQGQPGYYLTSPLQLGQGQQGYYPTSLQQPGQGQQPGQWQQSGQGQHWYYPTSPQLSGQGQRPGQWLQPGQGQQGYYPTSPQQPGQG
1D^{t}x-PI603223^{H10} GQQPGQGQQPGQGQPWYYPTSPQESGQGQQ------PGQWQQPGQGQPGYYLTSPLQLGQGQQGYYPTSLQQPGQGQQPGQWQQSGQGQHWYYPTSPQLSGQGQRPGQWLQPGQGQQGYYPTSPQQPGQG
1D^{t}x-IG46663^{H3} GQQPGQGQQPGQGQPWYYPTSPQESGQGQQ------PGQWQQPGQGQPGYYLTSPLQLGQGQQGYYPTSLQQPGQGQQPGQWQQSGQGQHGYYPTSPQLSGQGQRPGQWLQPGQGQQGYYPTSPQQSGQG
1D^{t}x-PI603224^{H4} GQQPGQGQQPGQGQPWYYPTSPQESEQGQQ------PGQWQQPGQGQPGYYLTSPLQLGQGQQGYYPTSLQQPGQGQQPGQWQQSGQGQHGYYPTSPQLSGQGQRPGQWLQPGQGQQGYYPTSPQQSGQG
1D^{t}x-IG48561^{H5} GQQPGQGQQPGQGQPWYYPTSPQESGQGQQ------PGQWQQPGQGQPGYYLTSPLQLGQGQQGYYPTSLQQPGQGQQPGQWQQSGQGQHGYYPTSPQLSGQGQRPGQWLQPGQGQQGYYPTSPQQSGQG
1D^{t}x-PI603236^{H6} ---------PGQGQPWYYPTSPQESGQGQQ------PGQWQQPGQGQPGYYLTSPLQLGQGQQGYYPTSLQQPGQGQQPGQWQQSGQGQHGYYPTSPQLSGQGQRPGQWLQPGQ---GYYPTSPQQSGQG
1D^{t}x-CIAE24^{H7} ---------PGQGQPWYYPTSPQESGQGQQ------PGQWQQPGQGQPGYYLTSPLQLGQGQQGYYPTSLQQPGQGQQPGQWQQSGQGQHGYYPTSPQLSGQGQRPGQWLQPGQGQQGYYPTSPQQSGQG
1D^{t}x-TA2527^{H8} ---------PGQGQPWYYPTSPQESGQGQQ------PGQWQQPGQGQPGYYLTSPLQLGQGQQGYYPTSLQQPGQGQQPGQWQQSGQGQHGYYPTSPQLSGQGQRPGQWLQPGQGQQGYYPTSPQQSGQG
1D^{t}x-PI511368^{H2} ---------PGQGQPWYYPTSPQESGQGQQPGQWQQPGQWQQPGQGQPGYYLTSPLQLGQGQQGYYPTSLQQPGQGQQPGQWQQSGQGQHGYYPTSPQLSGQGQRPGQWLQPGQGQQGYYPTSPQQSGQG
1D^{s}x-PI348360^{H2} ---------PGQGQPWYYPTSPQESGQGQQPGQWQQPGQWQQPGQGQPGYYLTSPLQLGQGQQGYYPTSLQQPGQGQQPGQWQQSGQGQHGYYPTSPQLSGQGQRPGQWLQPGQGQQGYYSTSPQQSGQG
1D^{s}x-TRI5008^{H11} ---------PGQGQPWYYPTSPQESGQGQQPGQWQQPGQWQQPGQGQPGYYLTSPLQLGQGQRGYYPTSLQQPGQGQQPGQWQQSGQGQHGYYPTSPQLSGQGQRPGQWLQPGQGQQGYYPTSPQQSGQG
1Dx2 ---------PGQGQPWYYPTSPQESGQGQQPGQWQQPGQWQQPGQGQPGYYLTSPLQLGQGQQGYYPTSLQQPGQGQQPGQWQQSGQGQHGYYPTSPQLSGQGQRPGQWLQPGQGQQGYYPTSPQQSGQG
****:*********** ****   *************************:*************************** ***:******************   ***.*****.***

1Dx5	QQLGQWLQPGQGQQGYYPTSLQQTGQGQQSGQGQQGYY***SSYHVSVEHQAASLKVAKAQQLAAQLPAMCRLEGGDALSASQ-***	848
1D^{s}x-PI15865^{H1}	QQLGQWLQPGQGQQGYYPTSLQQTGQGQQSGQGQQGYY***SSYHVSVEHQAASLKVAKAQQLAAQLPAMCRLEGGDALSASQ-***	848
1D^{s}x-PI361813^{H1}	QQLGQWLQPGQGQQGYYPTSLQQTGQGQQSGQGQQGYY***SSYHVSVEHQAASLKVAKAQQLAAQLPAMCRLEGGDALSASQ-***	848
1D^{s}x-TRI9883^{H1}	QQLGQWLQPGQGQQGYYPTSLQQTGQGQQSGQGQQGYY***SSYHVSVEHQAASLKVAKAQQLAAQLPAMCRLEGGDALSASQ-***	848
1D^{s}x-TRI16607^{H1}	QQLGQWLQPGQGQQGYYPTSLQQTGQGQQSGQGQQGYY***SSYHVSVEHQAASLKVAKAQQLAAQLPAMCRLEGGDALSASQ-***	848
1D^{s}x-TRI16981^{H1}	QQLGQWLQPGQGQQGYYPTSLQQTGQGQQSGQGQQGYY***SSYHVSVEHQAASLKVAKAQQLAAQLPAMCRLEGGDALSASQ-***	848
1D^{s}x-PI347904^{H1}	QQLGQWLQPGQGQQGYYPTSLQQTGQGQQSGQGQQGYY***SSYHVSVEHQAASLKVAKAQQLAAQLPAMCRLEGGDALSASQ-***	848
1D^{s}x-PI348150^{H1}	QQLGQWLQPGQGQQGYYPTSLQQTGQGQQSGQGQQGYY***SSYHVSVEHQAASLKVAKAQQLAAQLPAMCRLEGGDALSASQ-***	848
1D^{s}x-PI348171^{H1}	QQLGQWLQPGQGQQGYYPTSLQQTGQGQQSGQGQQGYY***SSYHVSVEHQAASLKVAKAQQLAAQLPAMCRLEGGDALSASQ-***	848
1D^{s}x-TRI19057^{H12}	QQLGQWLQPGQGQQGYYPTSLQQTGQGQQSGQGQQGYY***SSYHVSVEHQAASLKVAKAQQLAAQLPAMCRLEGGDALSASQ-***	839
1D^{t}x-PI349047^{H9}	QQLGQWLQPGQGQQGYYPTSLQRTGQGQQSGQGQQGYY***SSYHVSVEHQAASLKVAKAQQLAAQLPAMCRLEGGDALSASQG***	855
1D^{t}x-PI603223^{H10}	QQLGQWLQPGQGQQGYYPTSLQQTGQGQQSGQGQQGYY***SSYHVSVEHQAASLKVAKAQQLAAQLPAMCRLEGDDALSASQG***	855
1D^{t}x-IG46663^{H3}	QQLGQWLQPGQGQQGYYPTSLQQTGQGQQSGQGQQGYY***SSYHVSVEHQAASLKVAKAQQLAAQLPAMCRLEGGDALSASQ-***	845
1D^{t}x-PI603224^{H4}	QQLGQWLQPGQGQQGYYPTSLQQTGQGQQSGQGQQGYY***SSYHVSVEHQAASLKVAKAQQLAAQLPAMCRLEGGDALSASQ-***	845
1D^{t}x-IG48561^{H5}	QQLGQWLQPGQGQQGYYPTSLQQTGQGQQSGQGQQGYY***SSYHVSVEHQAASLKVAKAQQLAAQLPAMCRLEGGDALSASQ-***	845
1D^{t}x-PI603236^{H6}	QQLGQWLQPGQGQQGYYPTSLQQTGQGQQSGQGQQGYY***SSYHVSVEHQAASLKVAKAQQLAAQLPAMCRLEGGDALSASQ-***	833
1D^{t}x-CIAE24^{H7}	QQLGQWLQPGQGQQGYYPTSLQQTGQGQQSGQGQQGYY***SSYHVSVEHQAASLKVAKAQQLAAQLPAMCRLEGGDALSASQ-***	845
1D^{t}x-TA2527^{H8}	QQLGQWLQPGQGQQGYYPTSLQQTGQGQQSGQGQQGYY***SSYHVSVEHQAASLKVAKAQQLAAQLPAMCRLEGGDALSASQ-***	845
1D^{t}x-PI511368^{H2}	QQLGQWLQPGQGQQGYYPTSLQQTGQGQQSGQGQQGYY***SSYHVSVEHQAASLKVAKAQQLAAQLPAMCRLEGGDALSASQ-***	842
1D^{s}x-PI348360^{H2}	QQLGQWLQPGQGQQGYYPTSLQQTGQGQQSGQGQQGYY***SSYHVSVEHQAASLKVAKAQQLAAQLPAMCRLEGGDALSASQ-***	839
1D^{s}x-TRI5008^{H11}	QQLGQWLQPGQGQQGYYPTSLQQTGQGQQSGQGQQGYY***SSYHVSVEHQAASLKVAKAQQLAAQLPAMCRLEGGDALSASQ-***	839
1Dx2	QQLGQWLQPGQGQQGYYPTSLQQTGQGQQSGQGQQGYY***SSYHVSVEHQAASLKVAKAQQLAAQLPAMCRLEGGDALSASQ-***	839
	**********************:***	

Figure S4 Multiple alignment of the deduced amino acid sequences of 1Dx5 and 1Dx2 subunits from common wheat, and the 20 1Dx subunits from *Ae. tauschii* and *T. spelta*. The signal peptide is underlined, while the N- and C-terminal domains are labeled bold and bold italic, respectively. The repetitive domain is situated between the N- and C-terminal domains. The amino acid substitutions (S1 to S10) and indels (ID1 to ID5) between 1Dx5 and 1Dx2, and their variations in the 20 1Dx subunits from *T. spelta* and *Ae. tauschii*, are indicated. The four cysteine residues conserved among the 22 compared 1Dx subunits are marked by arrowheads. In addition, the cysteine reside unique to 1D^{s}x-TRI9883^{H1}, 1D^{s}x-TRI16607^{H1} or 1D^{s}x-TRI5008^{H11} is boxed.
