# Supplementary material for: Haplotype Variation of Glu-D1 Locus and the Origin of Glu-D1d Allele Conferring Superior End-Use Qualities in Common Wheat
Source: PLoS One. 2013 Sep 30;8(9):e74859. doi: 10.1371/journal.pone.0074859 (PMC3786984; doi:10.1371/journal.pone.0074859)
Supplement: Figure S5 — Detection of 1Dy genes in the two common wheat varieties, Bobwhite (harboring 1Dy10 ) and Chinese Spring (CS, containing 1Dy12 ), and the T. spelta and Ae. tauschii accessions with different Glu-D1 haplotypes using the PCR marker UMN26. This marker is co-dominant for 1Dy10 and 1Dy12, and allows the distinction between the two genes. Lane M contains DNA size standard (bp). (PDF) [file pone.0074859.s005.pdf]

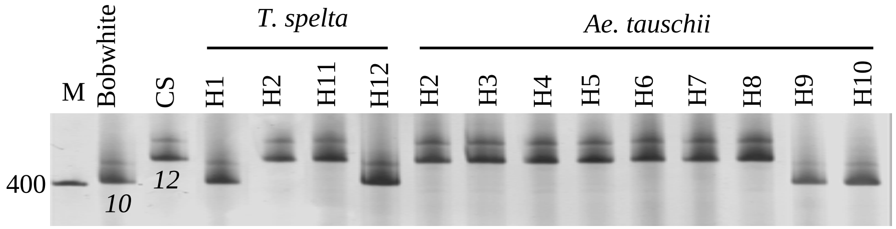

Figure S5 Detection of *1Dy* genes in the two common wheat varieties, Bobwhite (harboring *1Dy10*) and Chinese Spring (CS, containing *1Dy12*), and the *T. spelta* and *Ae. tauschii* accessions with different *Glu-D1* haplotypes using the PCR marker UMN26. This marker is co-dominant for *1Dy10* and *1Dy12*, and allows the distinction between the two genes. Lane M contains DNA size standard (bp).
