# Supplementary material for: Haplotype Variation of Glu-D1 Locus and the Origin of Glu-D1d Allele Conferring Superior End-Use Qualities in Common Wheat
Source: PLoS One. 2013 Sep 30;8(9):e74859. doi: 10.1371/journal.pone.0074859 (PMC3786984; doi:10.1371/journal.pone.0074859)
Supplement: Figure S6 — Multiple alignment of the amino acid sequences of 1Dy10 and 1Dy12 subunits from common wheat and the five 1Dy subunits from Ae. tauschii and T. spelta . The signal peptide is underlined, while the N- and C-terminal domains are labeled bold and bold italic, respectively. The repetitive domain resides between the N- and C-terminal domains. The amino acid substitutions (S1 to S12) and indels (ID1 to ID3) patterns between 1Dy10 and 1Dy12, and their variations in the five 1Dy subunits from Ae. tauschii, are indicated. The seven cysteine residues conserved among the seven compared 1Dy subunits are labeled by arrowheads. (PDF) [file pone.0074859.s006.pdf]

```
                                                  ▼          ▼                                ▼▼        ▼
1Dy10             MAKRLVLFAAVVIALVALTTAEGEASRQLQCERELQESSLEACRQVVDQQLAGRLPWSTGLQMRCCQQLRDVSAKCRSVAVSQVARQYEQTVVPPKGGSFYPGETTPLQQLQQGIFWGTS
1Dsy-TRI19057H12  MAKRLVLFAAVVIALVALTTAEGEASRQLQCERELQESSLEACRQVVDQQLAGRLPWSTGLQMRCCQQLRDVSAKCRSVAVSQVARQYEQTVVPPKGGSFYPGETTPLQQLQQGIFWGTS
1Dty-PI603223H10  MAKRLVLFAAVVIALVALTTAEGEASRQLQCERELQESSLEACRQVVDQQLAGRLPWSTGLQMRCCQQLRDVSAKCRSVAISQVARQYEQTVVPPKGGSFYPGETTPLQQLQQGIFWGTS
1Dty-IG4856H5     MAKRLVLFAAVVIALVALTTAEGEASRQLQCERELQESSLEACRQVVDQQLAGRLPWSTGLQMRCCQQLRDVSAKCRSVAVSQVARQYEQTVVPPKGGSFYPGETTPLQQLQQGIFWGTS
1Dty-PI511368H2   MAKRLVLFAAVVIALVALTTAEGEASRQLQCERELQESSLEACRQVVDQQLAGRLPWSTGLQMRCCQQLRDVSAKCRSVAVSQVARQYEQTVVPPKGGSFYPGETTPLQQLQQGIFWGTS
1Dsy-PI348360H2   MAKRLVLFAAVVIALVALTTAEGEASRQLQCERELQESSLEACRQVVDQQLAGRLPWSTGLQMRCCQQLRDVSAKCRSVAVSQVARQYEQTVVPPKGGSFYPGETTPLQQLQQGIFWGTS
1Dy12             MAKRLVLFAAVVIALVALTTAEGEASRQLQCERELQESSLEACRQVVDQQLAGRLPWSTGLQMRCCQQLRDVSAKCRSVAVSQVARQYEQTVVPPKGGSFYPGETTPLQQLQQGIFWGTS
                  ********************************************************************************:***************************************

                           S1                                                      S2                     ID1       S3                        ID2
1Dy10             SQTVQGYYPGVTSPRQGSYYPGQASPQQPGQGQQPGKWQEPGQGQQWYYPTSLQQPGQGQQIGKGQQGYYPTSLQQPGQGQQ------GYYPTSLQHTGQRQQPVQGQQ------PEQGQ
1Dsy-TRI19057H12  SQTVQGYYPGVTSPRQGSYYPGQASPQQPGQGQQPGKWQEPGQGQQWYYPTSLQQPGQGQQIGKGQQGYYPTSLQQPGQGQQ------GYYPTSLQHTGQRQQPVQGQQ------PEQGQ
1Dty-PI603223H10  SQTVQGYYPGVTSPRQGSYYPGQASPQQPGQGQQPGKWQEPGQGQQWYYPTSLQQPGQGQQIGKGQQGYYPTSLQQPGQGQQ------GYYPTSLQHTGQRQQPVQGQQ------PEQGQ
1Dty-IG4856H5     SQTVQGYYPSVTSPRQGSYYPGQASPQQPGQGQQPGKWQEPGQGQQWYYPTSLQQPGQGQQIGKGQQGYYPTSLQQPGQGQQIGQGQQGYYPTSPQHTGQRQQPVQGQQIGQGQQPEQEQ
1Dty-PI511368H2   SQTVQGYYPSVTSPRQGSYYPGQASPQQPGQGQQPGKWQEPGQGQQWYYPTSLQQPGQGQQIGKGKQGYYPTSLQQPGQGQQIGQGQQGYYPTSPQHTGQRQQPVQGQQIGQGQQPEQGQ
1Dsy-PI348360H2   SQTVQGYYPSVTSPRQGSYYPGQASPQQPGQGQQPGKWQEPGQGQQWYYPTSLQQPGQGQQIGKGKQGYYPTSLQQPGQGQQIGQGQQGYYPTSPQHTGQRQQPVQGQQIGQGQQPEQGQ
1Dy12             SQTVQGYYPSVTSPRQGSYYPGQASPQQPGQGQQPGKWQEPGQGQQWYYPTSLQQPGQGQQIGKGKQGYYPTSLQQPGQGQQIGQGQQGYYPTSPQHTGQRQQPVQGQQIGQGQQPEQGQ
                  *********.*******************************************************:****************      ****** **************      *** *

                                          S4                                   S5
1Dy10             QPGQWQQGYYPTSPQQLGQGQQPRQWQQSGQGQQGHYPTSLQQPGQGQQGHYLASQQQPGQGQQGHYPASQQQPGQGQQGHYPASQQQPGQGQQGHYPASQQEPGQGQQGQIPASQQQPG
1Dsy-TRI19057H12  QPGQWQQGYYPTSPQQLGQGQQPRQWQQSGQGQQGHYPTSLQQPGQGQQGHYLASQQQPGQGQQGHYPASQQQPGQGQQGHYPASQQQPGQGQQGHYPASQQEPGQGQQGQIPASQQQPG
1Dty-PI603223H10  QPGQWQQGYYPTSPQQLGQGQQPGQWQQSGQGQQGHYPTSLQQPGQGQQGHYLASQQQPGQGQQGHYPASQQQPGQGQQGHYPASQQQPGQGQQGHYPASQQEPGQGQQGQIPASQQQPG
1Dty-IG4856H5     QPGQWQQGYYPTSPQQLGQGQQPGQWQQSGQGQQGHYPTSLQQPGQGQQGHYLASQQQPGQGQQGHYPASQQQPGQGQQGHYPASQQQPGQGQQGHYPASQQEPGQGQQGQIPASQQQPG
1Dty-PI511368H2   QPGQWQQGYYPTSPQQLGQGQQPGQWQQSGQGQQGHYPTSLQQPGQGQQGHYLASQQQPAQGQQGHYPASQQQPGQGQQGHYPASQQQPGQGQQGHYPASQQEPGQGQQGQIPASQQQPG
1Dsy-PI348360H2   QPGQWQQGYYPTSPQQLGQGQQPGQWQQSGQGQQGHYPTSLQQPGQGQQGHYLASQQQPAQGQQGHYPASQQQPGQGQQGHYPASQQQPGQGQQGHYPASQQEPGQGQQGQIPASQQQPG
1Dy12             QPGQWQQGYYPTSPQQLGQGQQPGQWQQSGQGQQGHYPTSLQQPGQGQQGHYLASQQQPAQGQQGHYPASQQQPGQGQQGHYPASQQQPGQGQQGHYPASQQEPGQGQQGQIPASQQQPG
                  *********************** ***********************************.************************************************************

                                ID3             S6
1Dy10             QGQQGHYPASLQQPGQGQQGHYPTSLQQLGQGQQTGQPGQKQQPGQGQQTGQGQQPEQEQQPGQGQQGYYPTSLQQPGQGQQQGQGQQGYYPTSLQQPGQGQQGHYPASLQQPGQGQPGQ
1Dsy-TRI19057H12  QGQQGHYPASLQQPGQGQQGHYPTSLQQLGQGQQTGQPGQKQQPGQGQQTGQGQQPEQEQQPGQGQQGYYPTSLQQPGQGQQQGQGQQGYYPTSLQQPGQGQQGHYPASLQQPGQGQPGQ
1Dty-PI603223H10  QGQQGHYPASLQQPGQGQQGHYPTSLQQLGQGQQTGQPGQKQQPGQGQQTGQGQQPEQEQQPGQGQQGYYPTSLQQPGQGQQQGQGQQGYYPTSLQQPGQGQQGHYPASLQQPGQGQPGQ
1Dty-IG4856H5     QGQQGHYPASLQQPGQGQQGHYPTSLQQLGQGQQIGQPGQKQQPGQGQQTGQGQQPEQEQQPGQGQQGYYPTSLQQPGQGQQQGQGQQGYYPTSLQQPGQGQQGHYPASLQQPEQGQPGQ
1Dty-PI511368H2   QGQQGHYPASLQQP--GQQGHYPTSLQQLGQGQQIGQPGQKQQPGQGQQTGQGQQPEQEQQPGQGQQGYYPTSLQQPGQGQQQGQGQQGYYPTSLQQPGQGQQGHYPASLQQPGQGQPGQ
1Dsy-PI348360H2   QGQQGHYPASLQQP--GQQGHYPTSLQQLGQGQQIGQPGQKQQPGQGQQTGQGQQPEQEQQPGQGQQGYYPTSLQQPGQGQQQGQGQQGYYPTSLQQPGQGQQGHYPASLQQPGQGQPGQ
1Dy12             QGQQGHYPASLQQP--GQQGHYPTSLQQLGQGQQIGQPGQKQQPGQGQQTGQGQQPEQEQQPGQGQQGYYPTSLQQPGQGQQQGQGQQGYYPTSLQQPGQGQQGHYPASLQQPGQGQPGQ
                  **************  ****************** ****************************************************************************** ******

                                S7                                                         ▼S8    S9     S10                          S11
1Dy10             RQQPGQGQHPEQGKQPGQGQQGYYPTSPQQPGQGQQLGQGQQGYYPTSPQQPGQGQQPGQGQQGHCPTSPQQSGQAQQPGQGQQIGQVQQPGQGQQGYYPTSVQQPGQGQQSGQGQQSGQ
1Dsy-TRI19057H12  RQQPGQGQHPEQGKQPGQGQQGYYPTSPQQPGQGQQLGQGQQGYYPTSPQQPGQGQQPGQGQQGHCPTSPQQSGQAQQPGQGQQIGQVQQPGQGQQGYYPTSVQQPGQGQQSGQGQQSGQ
1Dty-PI603223H10  RQQPGQGQHPEQGKQPEQGQQGYYPTSPQQPGQGQQLGQGQQGYYPTSPQQPGQGQQPGQGQQGHCPTSPQQSGQAQQPGQGQQIGQVQQPGQGQQGYYPTSLQQPGQGQQSGQGQQSGQ
1Dty-IG4856H5     RQQPGQGQHPEQGQQPGQGQQGYYPTSPQQPGQGQQLGQGQQGYYPTSPQQPGQGQQ------GHCPTSPQQTGQAQQPGQGQQIGQVQQPGQGQQGYYPTSLQQPGQGQQSGQGQQSGQ
1Dty-PI511368H2   RQQPGQGQHPEQGQQPGQGQQGYYPTSPQQPGQGQQLGQGQQGYYPTSPQQPGQGQQPGQGQQGHCPMSPQQTGQAQQLGQGQQIGQVQQPGQGQQGYYPTSLQQPGQGQQSGQGQQSGQ
1Dsy-PI348360H2   RQQPGQGQHPEQGQQPGQGQQGYYPTSPQQPGQGQQLGQGQQGYYPTSPQQPGQGQQPGQGQQGHCPMSPQQTGQAQQLGQGQQIGQVQQPGQGQQGYYPTSLQQPGQGQQSGQGQQSGQ
1Dy12             RQQPGQGQHPEQGQQPGQGQQGYYPTSPQQPGQGQQLGQGQQGYYPTSPQQPGQGQQPGQGQQGHCPMSPQQTGQAQQLGQGQQIGQVQQPGQGQQGYYPTSLQQPGQGQQSGQGQQSGQ
                  *************:** ****************************************      **** ****:***** ***********************:*****************

                                S12                              ▼
1Dy10             GHQPGQGQQSGQEQQGYDSPYHVSAEQQAASPMVAKAQQPATQLPTVCRMEGGDALSASQ   648
1Dsy-TRI19057H12  GHQPGQGQQSGQEQQGYDSPYHVSAEQQAASPMVAKAQQPATQLPTVCRMEGGDALSASQ   648
1Dty-PI603223H10  GHQPGQGQQSGQEQQGYDSPYHVSAEQQAASPMVAKAQQPATQLPTVCRMEGGDALSASQ   648
1Dty-IG4856H5     GHQPGQRQQLGQEQQGYDSPYHVSAEQQAASPMVAKAQQPATQLSTACRMEGGDALSASQ   654
1Dty-PI511368H2   GHQPGQGQQSGQEQQGYDSPYHVSAEQQAASPMVAKAQQPATQLPTVCRMEGGDALSASQ   658
1Dsy-PI348360H2   GHQPGQGQQSGQEKQGYDSPYHVSAEQQAASPMVAKAQQPATQLPTVCRMEGGDALSASQ   658
1Dy12             GHQPGQGQQSGQEKQGYDSPYHVSAEQQAASPMVAKAQQPATQLPTVCRMEGGDALSASQ   658
                  ****** ** ***:******************************.*.*************
```

Figure S6 Multiple alignment of the amino acid sequences of 1Dy10 and 1Dy12 subunits from common wheat and the five 1Dy subunits from *Ae. tauschii* and *T. spelta*. The signal peptide is underlined, while the N- and C-terminal domains are labeled bold and bold italic, respectively. The repetitive domain resides between the N- and C-terminal domains. The amino acid substitutions (S1 to S12) and indels (ID1 to ID3) patterns between 1Dy10 and 1Dy12, and their variations in the five 1Dy subunits from *Ae. tauschii*, are indicated. The seven cysteine residues conserved among the seven compared 1Dy subunits are labeled by arrowheads.
