# Supplementary material for: Haplotype Variation of Glu-D1 Locus and the Origin of Glu-D1d Allele Conferring Superior End-Use Qualities in Common Wheat
Source: PLoS One. 2013 Sep 30;8(9):e74859. doi: 10.1371/journal.pone.0074859 (PMC3786984; doi:10.1371/journal.pone.0074859)
Supplement: Figure S7 — A diagram illustrating the variations of 1Dx and 1Dy genes and the alleles of the seven DNA markers in 12 Glu-D1 haplotypes. The types of 1Dx and 1Dy genes and the alleles of the seven Glu-D1 markers were indicated. (PDF) [file pone.0074859.s007.pdf]

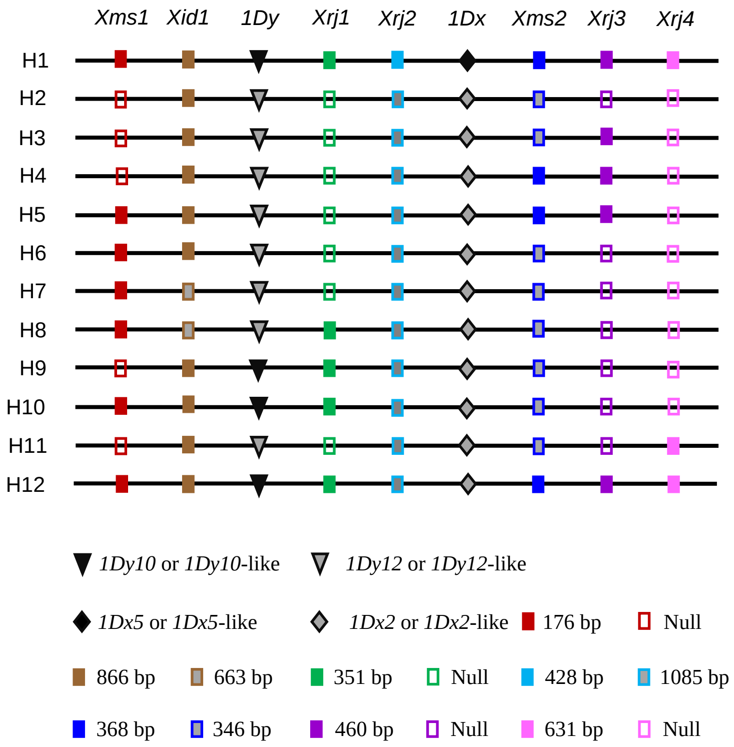

Figure S7 A diagram illustrating the variations of *1Dx* and *1Dy* genes and the alleles of the seven DNA markers in 12 *Glu-D1* haplotypes. The types of *1Dx* and *1Dy* genes and the alleles of the seven *Glu-D1* markers were indicated.
