# Supplementary material for: Haplotype Variation of Glu-D1 Locus and the Origin of Glu-D1d Allele Conferring Superior End-Use Qualities in Common Wheat
Source: PLoS One. 2013 Sep 30;8(9):e74859. doi: 10.1371/journal.pone.0074859 (PMC3786984; doi:10.1371/journal.pone.0074859)
Supplement: Figure S8 — A simplified model illustrating possible derivative relationships among 12 Glu-D1 haplotypes. The arrows indicate the likely directions of haplotype differentiation. The approximate differentiation times (MYA) of the haplotypes in Ae. tauschii and hexaploid wheat were indicated. The three haplotypes (H5, H10 and H12) involved in the differentiation of H1 are labeled in bold. H2, detected in both Ae. tauschii and hexaploid wheat, was shown in italic. (PDF) [file pone.0074859.s008.pdf]

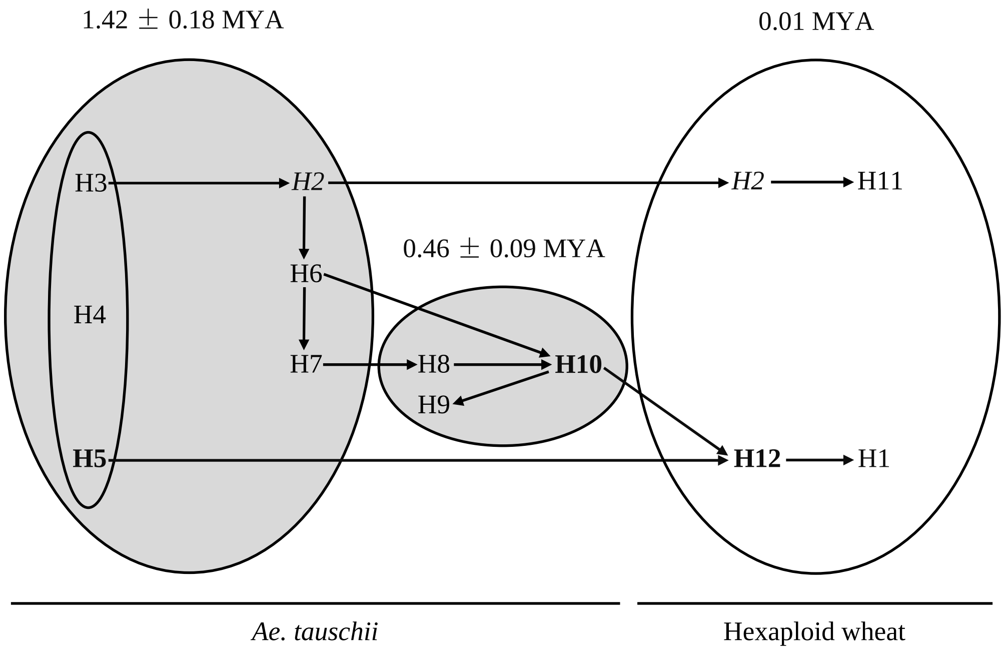

Figure S8 A simplified model illustrating possible derivative relationships among 12 *Glu-D1* haplotypes. The arrows indicate the likely directions of haplotype differentiation. The approximate differentiation times (MYA) of the haplotypes in *Ae. tauschii* and hexaploid wheat were indicated. The three haplotypes (H5, H10 and H12) involved in the differentiation of H1 are labeled in bold. H2, detected in both *Ae. tauschii* and hexaploid wheat, was shown in italic.
