# Supplementary material for: Haplotype Variation of Glu-D1 Locus and the Origin of Glu-D1d Allele Conferring Superior End-Use Qualities in Common Wheat
Source: PLoS One. 2013 Sep 30;8(9):e74859. doi: 10.1371/journal.pone.0074859 (PMC3786984; doi:10.1371/journal.pone.0074859)
Supplement: Figure S9 — A diagram illustrating the positions of polymorphic sites among the 1Dx5 subunits from two common wheat varieties (Cheyenne and Renan) and the eight 1Dx5-like subunits from T. spelta genotypes. 1Dx5 protein sequence (GenBank accession CAA31395) from Cheyenne was used as reference for locating the positions of polymorphic sites, where amino acid substitutions were detected. The diagram is not drawn in scale. (PDF) [file pone.0074859.s009.pdf]

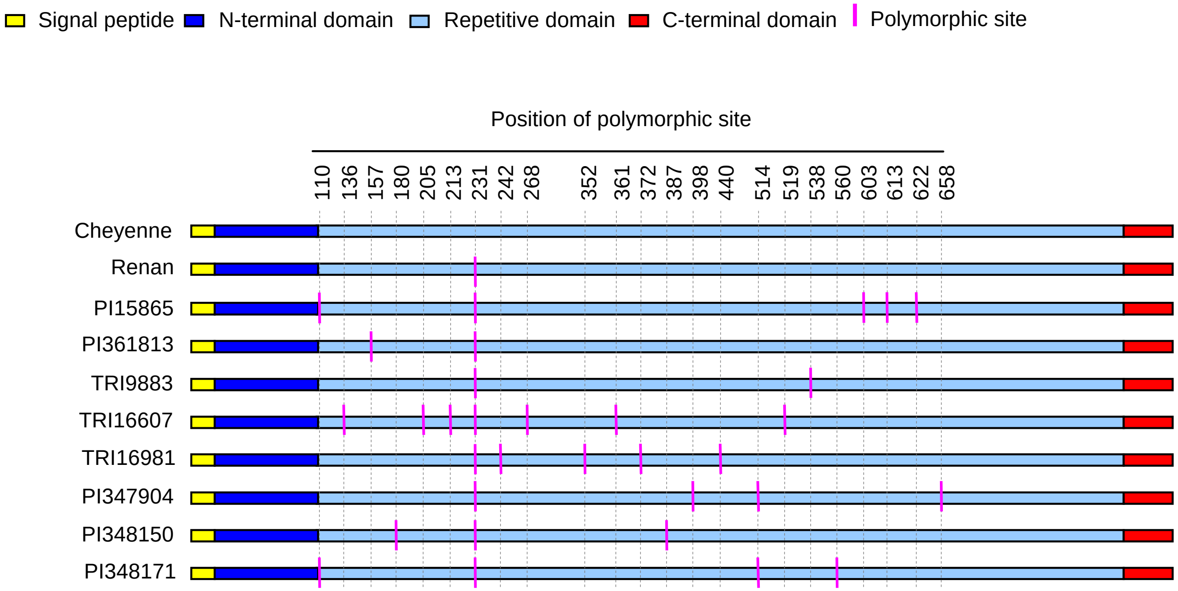

Figure S9 A diagram illustrating the positions of polymorphic sites among the 1Dx5 subunits from two common wheat varieties (Cheyenne and Renan) and the eight 1Dx5-like subunits from *T. spelta* genotypes. 1Dx5 protein sequence (GenBank accession CAA31395) from Cheyenne was used as reference for locating the positions of polymorphic sites, where amino acid substitutions were detected. The diagram is not drawn in scale.
