# Supplementary material for: Haplotype Variation of Glu-D1 Locus and the Origin of Glu-D1d Allele Conferring Superior End-Use Qualities in Common Wheat
Source: PLoS One. 2013 Sep 30;8(9):e74859. doi: 10.1371/journal.pone.0074859 (PMC3786984; doi:10.1371/journal.pone.0074859)
Supplement: Table S5 — The oligonucleotide primers used in this study. (DOC) [file pone.0074859.s014.doc]

**Table S5** The oligonucleotide primers used in this study

| Name | Sequence (5'-3') | Use | Product size (bp) |
| --- | --- | --- | --- |
| Xms1-F | TCAAGGGAATCAACTCAACC | Amplifying *Xms1* | 176 or null |
| Xms1-R | AACAAACCCAAACCGGACA |
| Xms2-F | TTTGTCAGTACTGGCCTACTTG | Amplifying *Xms2* | 346 or 368 |
| Xms2-R | ATGTGTGTAAAGTTGGTGAGAT |
| Xid1-F | CAGTTGTCGCCTTTTCCACA | Amplifying *Xid1* | 866 |
| Xid1-R | TGCTGCCAACAATCTTATCG |
| Xrj1-F | GGGCATATTTCCAACAAAAGTT | Amplifying *Xrj1* | 351 |
| Xrj1-R | AAAGGTCGCCATCACAAGAG |
| Xrj2-F | CTCGCTCGACGGTGTTGAA | Amplifying *Xrj2* | 428 or 1085 |
| Xrj2-R | CCTTTGGCCCAGATAAAGTG |
| Xrj3-F | CGTCATAACTTTGCGCTTGT | Amplifying *Xrj3* | 460 or null |
| Xrj3-R | GGGTCTAGATTGTAGCCTGCT |
| Xrj4-F | GAAGGGAAGCAGCCCTTAGT | Amplifying *Xrj4* | 631 or null |
| Xrj4-R | GAGATTGAACTAGGTATGTGCT |
| 1Dx-F | ATGGCTAAGCGGTTAGTC | Amplifying *1Dx* genes | 2536 - 2581 |
| 1Dx-R | GCTGCAGAGAGTTCTATC |
| 1Dy-F | CCACCGAGATGGCTAAGCGGC | Amplifying *1Dy* genes | 1968 - 1998 |
| 1Dy-R | GCAGAGAGTTCTATCACTGGCTA |
| Sal-F | CACAA/GGCCATTGATCTATGAT | Amplifying the left LTR of *Sabrina-2* | 1790 |
| Sal-R | AAGCAAGTGAAGAAGACAAACAG |
| Sar-F | GTATTGCGTGAAAGTTGAA | Amplifying the right LTR of *Sabrina-2* | 1967 |
| Sar-R | GCATATCTATGTTGTAGATTGCT |
