# Supplementary material for: Haplotype Variation of Glu-D1 Locus and the Origin of Glu-D1d Allele Conferring Superior End-Use Qualities in Common Wheat
Source: PLoS One. 2013 Sep 30;8(9):e74859. doi: 10.1371/journal.pone.0074859 (PMC3786984; doi:10.1371/journal.pone.0074859)
Supplement: Table S6 — Estimation of approximate insertion time of Sabrina-2 into Glu-D1 locus. (DOC) [file pone.0074859.s015.doc]

**Table S6** Estimation of approximate insertion time of *Sabrina-2* into *Glu-D1* locus

| Haplotype | Species | LTR length aligned  (bp) | Number of nucleotide substitutions | Approximate insertion time (MYA) |
| --- | --- | --- | --- | --- |
| H1 (Renan *Glu-D1*) | *T. aestivum* | 1575 | 76 | 2.01 ± 0.25 |
| H7 (AUS18913 *Glu-D1*) | *Ae. tauschii* | 1573 | 98 | 2.30 ± 0.22 |
