# Supplementary material for: Haplotype Variation of Glu-D1 Locus and the Origin of Glu-D1d Allele Conferring Superior End-Use Qualities in Common Wheat
Source: PLoS One. 2013 Sep 30;8(9):e74859. doi: 10.1371/journal.pone.0074859 (PMC3786984; doi:10.1371/journal.pone.0074859)
Supplement: Table S7 — Number of nucleotide substitutions between the LTR sequences in the Sabrina-2 elements resided in several Glu-D1 haplotypes. (DOC) [file pone.0074859.s016.doc]

**Table S7** Number of nucleotide substitutions between the LTR sequences in the *Sabrina-2* elements resided in several *Glu-D1* haplotypes

| Haplotype compared | Total LTR length aligned  (bp)^a^ | Number of nucleotide substitutions |
| --- | --- | --- |
| H1 *vs* H12 | 3152 | 0 |
| H1 *vs* H5 | 3148 | 32 |
| H1 *vs* H10 | 3152 | 16 |

^a^ Total LTR length refers to the combined size of the left and right long terminal repeats of *Sabrina-2*.
