# Supplementary material for: Haplotype Variation of Glu-D1 Locus and the Origin of Glu-D1d Allele Conferring Superior End-Use Qualities in Common Wheat
Source: PLoS One. 2013 Sep 30;8(9):e74859. doi: 10.1371/journal.pone.0074859 (PMC3786984; doi:10.1371/journal.pone.0074859)
Supplement: Table S8 — Amino acid substitutions (S1 to S10) and indels (ID1 to ID5) between 1Dx5 and 1Dx2 of common wheat and their variations in the 20 1Dx subunits from T. spelta or Ae. Tauschii. (DOC) [file pone.0074859.s017.doc]

**Table S8** Amino acid substitutions (S1 to S10) and indels (ID1 to ID5) between 1Dx5 and 1Dx2 of common wheat and their variations in the 20 1Dx subunits from *T. spelta* or *Ae. tauschii*

| 1Dx | S1  (118)^a^ | S2 (167) | S3  (176) | S4  (195) | S5  (231) | S6  (331) | S7  (427) | S8  (584) | S9  (729) | S10  (765) | ID1  (145-146) | ID2  (591-599) | ID3  (618-620) | ID4  (645-653) | ID5  (674-675) |
| --- | --- | --- | --- | --- | --- | --- | --- | --- | --- | --- | --- | --- | --- | --- | --- |
| 1Dx5 | C | R | S | Q | A | G | Q | R | W | P | - | 9 | 3 | 9 | - |
| **1D^s^x-PI15865****^H1^** | C | R | S | Q | G | G | Q | R | W | P | - | 9 | 3 | 9 | - |
| **1D^s^x-PI361813^H1^** | C | R | S | Q | G | G | Q | R | W | P | - | 9 | 3 | 9 | - |
| **1D^s^x-TRI16607^H1^** | C | R | S | Q | G | G | Q | R | W | P | - | 9 | 3 | 9 | - |
| **1D^s^x-TRI16981^H1^** | C | R | S | Q | G | G | Q | R | W | P | - | 9 | 3 | 9 | - |
| **1D^s^x-TRI9883^H1^** | C | R | S | Q | G | G | Q | R | W | P | - | 9 | 3 | 9 | - |
| **1D^s^x-PI347904^H1^** | C | R | S | Q | G | G | Q | R | W | P | - | 9 | 3 | 9 | - |
| **1D^s^x-PI348150^H1^** | C | R | S | Q | G | G | Q | R | W | P | - | 9 | 3 | 9 | - |
| **1D^s^x-PI348171^H1^** | C | R | S | Q | G | G | Q | R | W | P | - | 9 | 3 | 9 | - |
| 1D^s^x-TRI19057^H12^ | S | R | S | Q | G | G | Q | R | W | P | - | 9 | 3 | - | - |
| 1D^t^x-PI349047^H9^ | S | G | S | Q | G | G | Q | R | W | P | 6 | 9 | 3 | 9 | - |
| 1D^t^x-PI603223^H10^ | S | G | S | Q | G | G | Q | R | W | P | 6 | 9 | 3 | 9 | - |
| 1D^t^x-IG48561^H5^ | S | G | P | Q | G | G | Q | Q | G | S | 6 | - | 3 | 9 | - |
| 1D^t^x-IG46663^H3^ | S | G | P | Q | G | G | Q | Q | G | S | 6 | - | 3 | - | - |
| 1D^t^x-PI603224^H4^ | S | G | P | Q | G | G | Q | Q | G | S | 6 | - | 3 | - | - |
| 1D^t^x-CIAE24^H7^ | S | G | P | R | G | G | Q | Q | G | S | 6 | 9 | 3 | - | - |
| 1D^t^x-TA2527^H8^ | S | G | P | R | G | G | Q | Q | G | S | 6 | 9 | 3 | - | - |
| 1D^t^x-PI603236^H6^ | S | G | P | R | G | G | Q | Q | G | S | 6 | - | 3 | - | - |
| 1D^t^x-PI511368^H2^ | S | G | P | R | G | E | L | Q | G | S | 6 | - | 3 | - | 6 |
| 1D^s^x-PI348360^H2^ | S | G | P | R | G | E | L | Q | G | S | 6 | - | - | - | 6 |
| 1D^s^x-TRI5008^H11^ | S | G | P | R | G | E | L | Q | G | S | 6 | - | - | - | 6 |
| 1Dx2 | S | G | P | R | G | E | L | Q | G | S | 6 | - | - | - | 6 |

The 1Dx5-like and 1Dx2-like subunits are shown in bold and underlined, respectively. The substitutions and indels identical to those of 1Dx5 are shaded in yellow, whereas those identical to 1Dx2 by blue.

^a^ The value in the brackets marks the position of the substitution or indel calculated with the protein sequence of 1Dx5 (GenBank accession CAA31395).
