# Supplementary material for: Haplotype Variation of Glu-D1 Locus and the Origin of Glu-D1d Allele Conferring Superior End-Use Qualities in Common Wheat
Source: PLoS One. 2013 Sep 30;8(9):e74859. doi: 10.1371/journal.pone.0074859 (PMC3786984; doi:10.1371/journal.pone.0074859)
Supplement: Table S9 — Divergence time estimates between two representative hexaploid wheat 1Dx genes ( 1Dx5 and 1Dx2 ) and Ae. tauschii 1Dx genes. (DOC) [file pone.0074859.s018.doc]

***Table S9*** *Divergence time estimates between two representative hexaploid wheat 1Dx genes (1Dx5 and 1Dx2) and Ae. tauschii 1Dx genes*

| *1Dx* from *Ae. tauschii* | | *1Dx5* | | *1Dx2* | |
| --- | --- | --- | --- | --- | --- |
|  |  | Divergence time (MY) | Average | Divergence time (MY) | Average |
| C1^a^ | *1D^t^x-PI349047^H9^* | 0.30 ± 0.08 | 0.29 | / |  |
|  | *1D^t^x-PI603223^H10^* | 0.28 ± 0.08 |  | / |  |
| C2 | *1D^t^x-PI511368^H2^* | / |  | 0.04 ± 0.02 | 0.13 |
|  | *1D^t^x-PI603236^H6^* | / |  | 0.14 ± 0.05 |  |
|  | *1D^t^x-CIAE24^H7^* | / |  | 0.16 ± 0.06 |  |
|  | *1D^t^x-TA2527^H8^* | / |  | 0.16 ± 0.06 |  |
| C3 | *1D^t^x-IG46663^H3^* | 0.40 ± 0.10 | 0.37 | 0.38 ± 0.09 | 0.36 |
|  | *1D^t^x-PI603224^H4^* | 0.35 ± 0.09 |  | 0.34 ± 0.09 |  |
|  | *1D^t^x-IG48561^H5^* | 0.36 ± 0.09 |  | 0.36 ± 0.09 |  |

^a^ Phylogenetic cluster (as detailed in Figure 2).
