# Supplementary material for: Haplotype Variation of Glu-D1 Locus and the Origin of Glu-D1d Allele Conferring Superior End-Use Qualities in Common Wheat
Source: PLoS One. 2013 Sep 30;8(9):e74859. doi: 10.1371/journal.pone.0074859 (PMC3786984; doi:10.1371/journal.pone.0074859)
Supplement: Methods S1 — Additional methods. (DOC) [file pone.0074859.s019.doc]

**Methods S1**

Plant materials

For validating the specificity of the newly developed *Glu-D1* DNA markers, the nulli-tetrasomic line N1DT1A and the ditelosomic line Dt1Ds of Chinese Spring [1],[2], two *T. turgidum* durum wheat varieties Langdon and Cofa, and two *Ae. tauschii* accessions (AUS18917, AL8/78) were included in addition to the common wheat varieties (Attila, Bobwhite, Chinese Spring, and Kenong 199). The germplasm lines used in this work were obtained mainly from National Small Grains Collection, US Department of Agriculture-Agricultural Research Service (Aberdeen, Idaho, USA), International Center for Agricultural Research in the Dry Areas (Aleppo, Syria), The Genebank of the Leibniz Institute of Plant Genetics and Crop Plant Research (Gatersleben, Germany), Henan University (Kaifeng, China), and Sichuan Agricultural University (Chengdu, China).

PCR and electrophoresis conditions

PCR was carried out in 20 μl volume containing 50 ng genomic DNA template, 10 mM dNTPs, 5 pmol of each primer, and 1 U Taq polymerase (Transgen Biotech, China). The cycling parameters were 94 °C for 5 min, followed by 33 cycles of 94 °C for 30 s, 60 to 62.5 °C for 30s and 72 °C for 1 min, and a final extension at 72 °C for 5 min. PCR products were separated in 1.5% agarose gels.

Cloning and constructing the nucleotide sequences of HMW-GS genes and LTR regions of *Sabrina-2* in *Glu-D1* locus

The PCR fragments of the expected size were recovered from agarose gels, followed by cloning into the pGEM-T Easy vector (Promega, Madison, USA) and DNA sequencing. Three separate clones derived from independent PCR amplifications and cloning trials were sequenced for constructing the final nucleotide sequence of each amplicon.

Sequence alignment and phylogenetic analysis

For comparing *1Dx* genes, the alignment was performed using both nucleotide and deduced amino acid sequences. As controls, the three well characterized common wheat *1Dx* genes, *1Dx5*, *1Dx2*, and *1Ax1* reported previously [3]-[5], were included in the alignment. The distance values among the aligned sequences were computed using the P distance model, and phylogenetic tree was constructed using the algorithms installed in MEGA 5.0 [6]. Bootstrap values were obtained based on 1000 permutations. For comparing *1Dy* genes, the alignment was conducted using deduced amino acid sequences. Two well studied common *1Dy* genes, *1Dy10* and *1Dy12* [4],[7], were included as controls.

**References**

1. Sears ER (1966) Nullisomic-tetrasomic combination in hexaploid wheat. In: Riley R, Lewis KR, editors. Chromosome Manipulation and Plant Genetics. Edinburgh: Oliver and Boyd. pp. 29-45.
2. Sears ER, Sears LMS (1978) The telocentric chromosomes of common wheat. In: Ramanujan S, editor. Proceedings of 5th International Wheat Genetics Symposium. New Delhi: Indian Agriculture Research Institute, 389-407.
3. Sugiyama T, Rafalski A, Paterson D, Soll D (1985) A wheat HMW glutenin subunit gene reveals a highly repeated structure. Nucl Acids Res 13: 8729-8737.
4. Anderson OD, Greene FC, Yip RE, Halford NG, Shewry PR, et al. (1989) Nucleotide sequences of the two high-molecular-weight glutenin genes from the D-genome of a hexaploid bread wheat, *Triticum aestivum* L. cv Cheyenne. Nucl Acids Res 17: 461-462.
5. Halford NG, Field JM, Blair H, Urwin P, Moore K, et al. (1992) Analysis of HMW glutenin subunits encoded by chromosome 1A of bread wheat (*Triticum aestivum* L.) indicates quantitative effects on grain quality. Theor Appl Genet 83: 373-378.
6. Tamura K, Peterson D, Peterson N, Stecher G, Nei M, et al. (2011) MEGA5: Molecular evolutionary genetics analysis using maximum likelihood, evolutionary distance, and maximum parsimony methods. Mol Biol Evol 28: 2731-2739.
7. Anderson OD (2009) EST mining for gene structure and expression analysis of genes in the region of the wheat high-molecular weight glutenin loci. Genome 52: 726-740.
