# Supplementary material for: Haplotype Variation of Glu-D1 Locus and the Origin of Glu-D1d Allele Conferring Superior End-Use Qualities in Common Wheat
Source: PLoS One. 2013 Sep 30;8(9):e74859. doi: 10.1371/journal.pone.0074859 (PMC3786984; doi:10.1371/journal.pone.0074859)
Supplement: Figure S2 — Multiple alignment of the DNA sequences of Xms2 amplicons from Attila, Bobwhite, Chinese Spring (CS) and Kenong 199, as well as the reference sequences from Renan and AUS18913. Chinese Spring (CS) and Kenong 199, as well as the reference sequences from Renan and AUS18913. The 22 bp deletion in the amplicons from CS, Kenong 199 and AUS18913 was indicated by dashed line. (PDF) [file pone.0074859.s002.pdf]

```
     Renan TTTGTCAGTACTGGCCTACTTGGGGGCCACTAGTGCAAGTAGTTTTATATGCTTGCATGCACATATGCTGTCGTTGAATTTGAGAACGGCGAGAAGGTGC
    Attila TTTGTCAGTACTGGCCTACTTGGGGGCCGCTAGTGCAAGTAGTTTTATATGCTTGCATGCACATATGCTGTCGTTGAATTTGAGAACGGCGAGAAGGTGC
  Bobwhite TTTGTCAGTACTGGCCTACTTGGGGGCCGCTAGTGCAAGTAGTTTTATATGCTTGCATGCACATATGCTGTCGTTGAATTTGAGAACGGCGAGAAGGTGC
        CS TTTGTCAGTACTGGCCTACTTGGGGGCCGCTAGTGCAAGTAGTTTTATATGCTTGCATGCACATATGCTGTCGTTGAATTTGAGAACGGCGAGAAGGTGC
Kenong 199 TTTGTCAGTACTGGCCTACTTGGGGGCCGCTAGTGCAAGTAGTTTTATATGCTTGCATGCACATATGCTGTCGTTGAATTTGAGAACGGCGAGAAGGTGC
  AUS18913 TTTGTCAGTACTGGCCTACTTGGGGGCCGCTAGTGCAGGTAGTTTTATATGCTTGCATGCACATATGCTGTCGTTGAATTTGAGAACGGCGAGAAGGTGC
           ****************************.********.**************************************************************

     Renan TCGCTCTTTAGTGACACTTGTGTGTGTGTGTGTGTGTGTGTGTGTGTGTGTGTGTGTGTGGCTAGAGTGCGTTGCAACAAAAGCGCAAAGTGAAGGGA
    Attila TCGCTCTTTAGTGACACTTGTGTGTGTGTGTGTGTGTGTGTGTGTGTGTGTGTGTGTGTGGCTAGAGTGCGTTGCAACAAAAGCGCAAAGTGAAGGGA
  Bobwhite TCGCTCTTTAGTGACACTTGTGTGTGTGTGTGTGTGTGTGTGTGTGTGTGTGTGTGTGTGGCTAGAGTGCGTTGCAACAAAAGCGCAAAGTGAAGGGA
        CS TCGCTCTTTAGTGACACTTGTGTGTGTGTGTGTGTGTGTG----------------------GCTAGAGTGCGTTGCAACAAAAGCGCAAAGTGAAGGGA
Kenong 199 TCGCTCTTTAGTGACACTTGTGTGTGTGTGTGTGTGTGTG----------------------GCTAGAGTGCGTTGCAACAAAAGCGCAAAGTGAAGGGA
  AUS18913 TCGCTCTTTAGTGACACTTGTGTGTGTGTGTGTGTGTGTG----------------------GCTAGAGTGCGTTGCAACAAAAGCGCAAAGTGAAGGGA
           ****************************************                      **************************************

     Renan TATGTGCTGCCGGGAAGCCCGGGGCAGTGTGGTTGGGAGGTGGATGGATGGATGGATATACACGAATGACGAACTTGCCTTTCATGCGTTTCTTTGCTTA
    Attila TATGTGCTGCCGGGAAGCCCGGGGCAGTGTGGTTGGGAGGTGGATGGATGGATGGATATACACGAATGACGAACTTGCCTTTCATGCGTTTCTTTGCTTA
  Bobwhite TATGTGCTGCCGGGAAGCCCGGGGCAGTGTGGTTGGGAGGTGGATGGATGGATGGATATACACGAATGACGAACTTGCCTTTCATGCGTTTCTTTGCTTA
        CS TATGTGCTGCCGGGAAGCCCGGGGCAGTGTGGTTGGGAGGTGGATGGATGGATGGATATACACGGATGACGAACTTGCCTTTCATGCGTTTCTTTGCTTA
Kenong 199 TATGTGCTGCCGGGAAGCCCGGGGCTGTGTGGTTGGGAGGTGGATGGATGGATGGATATACACGAATGACGAACTTGCCTTTCATGCGTTTCTTTGCTTA
  AUS18913 TATGTGCTGCCGGGAAGCCCGGGGCTGTGTGGTTGGGAGGTGGATGGATGGATGGATATACACGAATGACGAACTTGCCTTTCATGCGTTTCTTTGCTTA
           *************************:**************************************.***********************************

     Renan ACCCACTGGACTGGGTGCTGACCTGGAGTAGTTACGAGCCATGCATATCTCACCAACTTTACACACAT 368
    Attila ACCCACTGGACTGGGTGCTGACCTGGAGTAGTTACGAGCCATGCATATCTCACCAACTTTACACACAT 368
  Bobwhite ACCCACTGGACTGGGTGCTGACCTGGAGTAGTTACGAGCCATGCATATCTCACCAACTTTACACACAT 368
        CS ACCCACTGGACTGGGTGCTGACCTGGAGTAGTTACGAGCCATGCATATCTCACCAACTTTACACACAT 346
Kenong 199 ACCCACTGGACTGGGTGCTGACCTGGAGTAGTTACGAGCCATGCATATCTCACCAACTTTACACACAT 346
  AUS18913 ACCCACTGGACTGGGTGCTGACCTGGAGTAGTTACGAGCCATGCATATCTCACCAACTTTACACACAT 346
           ********************************************************************
```

Figure S2 Multiple alignment of the DNA sequences of *Xms2* amplicons from Attila, Bobwhite, Chinese Spring (CS) and Kenong 199, as well as the reference sequences from Renan and AUS18913. The 22 bp deletion in the amplicons from CS, Kenong 199 and AUS18913 was indicated by dashed line.
